# Pre‐rRNA Facilitates TopBP1‐Mediated DNA Double‐Strand Break Response

**DOI:** 10.1002/advs.202206931

**Published:** 2023-08-15

**Authors:** Di Xin, Xiaochen Gai, Yidi Ma, Zexing Li, Qilin Li, Xiaochun Yu

**Affiliations:** ^1^ School of Life Sciences Westlake University Hangzhou Zhejiang 310024 China; ^2^ Department of Hepatobiliary and Pancreatic Surgery and Zhejiang Provincial Key Laboratory of Pancreatic Disease The First Affiliated Hospital Zhejiang University School of Medicine Hangzhou Zhejiang 310003 China; ^3^ Westlake Laboratory of Life Sciences and Biomedicine Hangzhou Zhejiang 310024 China; ^4^ Institute of Basic Medical Sciences Westlake Institute for Advanced Study Hangzhou Zhejiang 310024 China; ^5^ School of Life Sciences Tianjin University Tianjin 300072 China

**Keywords:** ATR activation, DNA damage response, pre‐ribosomal RNA, TopBP1

## Abstract

In response to genotoxic stress‐induced DNA damage, TopBP1 mediates ATR activation for signaling transduction and DNA damage repair. However, the detailed molecular mechanism remains elusive. Here, using unbiased protein affinity purification and RNA sequencing, it is found that TopBP1 is associated with pre‐ribosomal RNA (pre‐rRNA). Pre‐rRNA co‐localized with TopBP1 at DNA double‐strand breaks (DSBs). Similar to pre‐rRNA, ribosomal proteins also colocalize with TopBP1 at DSBs. The recruitment of TopBP1 to DSBs is suppressed when cells are transiently treated with RNA polymerase I inhibitor (Pol I‐i) to suppress pre‐rRNA biogenesis but not protein translation. Moreover, the BRCT4‐5 of TopBP1 recognizes pre‐rRNA and forms liquid–liquid phase separation (LLPS) with pre‐rRNA, which may be the molecular basis of DSB‐induced foci of TopBP1. Finally, Pol I‐i treatment impairs TopBP1‐associated cell cycle checkpoint activation and homologous recombination repair. Collectively, this study reveals that pre‐rRNA plays a key role in the TopBP1‐dependent DNA damage response.

## Graphical abstract

Following the DSBs, pre‐rRNA mediates the recruitment of TopBP1 to DNA lesions. The BRCT4‐5 domain of TopBP1 recognizes pre‐rRNA at DNA lesions and forms liquid–liquid phase separation (LLPS) with pre‐rRNA, which activates ATR‐dependent signaling pathway and facilitates homologous recombination (HR) repair. Thus, pre‐rRNA plays a key role in the TopBP1‐dependent DNA damage response.

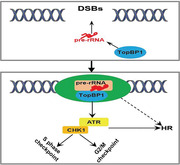

## 1 Introduction

DNA double‐strand break (DSB) is the most deleterious type of DNA lesions that easily induces genomic instability and tumorigenesis.^[^ ^1^, ^2^, ^3^ ^]^ Once DSBs occur, a group of PI3‐like kinases, including ATM, ATR, and DNA‐PK, are activated to arrest the cell cycle and facilitate DSB repair.^[^ ^4^ ^]^ Compared to the activation of ATM and DNA‐PK, activation of ATR requires several key signal transducers, and one of them is TopBP1.^[^ ^5^, ^6^ ^]^

TopBP1 is a 1522‐residue polypeptide with nine BRCT domains and an ATR activation domain (AAD).^[^ ^7^ ^]^ Although the AAD is a flexible coiled‐coil motif, it has been indicated that the ADD recognizes the ATR–ATRIP complex as well as the RAD9‐RAD1‐HUS1 (9‐1‐1) complex to facilitate ATR activation.^[^ ^6^, ^8^, ^9^ ^]^ However, the detailed molecular mechanism remains elusive. Nevertheless, DNA damage‐induced ATR activation is impaired when cells lack TopBP1.^[^ ^6^ ^]^ Once ATR is activated, ATR phosphorylates a number of substrates to induce cell cycle checkpoint activation and arrest cell cycle at S/G2 phase to facilitate DSB repair.^[^ ^10^, ^11^, ^12^, ^13^, ^14^, ^15^, ^16^ ^]^ The major downstream effector of the ATR signaling pathway is CHK1, a kinase that further phosphorylates CDC25A for the suppression of cyclin‐dependent kinases (CDKs).^[^ ^10^, ^11^, ^12^, ^13^, ^14^, ^15^, ^16^ ^]^ Hence, in response to DSBs, the ATR‐CHK1‐CDC25A axis is an important pathway to arrest the cell cycle at the S/G2 phase for DSB repair.^[^ ^10^, ^11^, ^12^, ^13^, ^14^, ^15^, ^16^ ^]^

In response to DSBs, TopBP1 is recruited to DNA lesions very quickly. It co‐localizes with other DNA damage response factors, such as γH2AX, and forms nuclear foci.^[^ ^17^, ^18^ ^]^ This interesting cellular phenomenon is dependent on the BRCT4‐5 of TopBP1.^[^ ^17^ ^]^ The BRCT domain is known as a phospho‐Ser binding motif.^[^ ^19^, ^20^, ^21^, ^22^ ^]^ And it has been shown that the BRCT4‐5 recognizes pSer motifs in BLM and 53BP1.^[^ ^18^, ^23^, ^24^, ^25^, ^26^, ^27^ ^]^ However, lacking BLM or 53BP1 does not affect the foci formation of TopBP1, nor the activation of ATR, indicating that the BRCT4‐5 recognizes other partners at DNA lesions for the foci formation of TopBP1.^[^ ^28^, ^29^, ^30^, ^31^, ^32^ ^]^

The DNA damage‐induced foci formation is a membraneless organelle in the nucleus for DSB repair.^[^ ^33^, ^34^ ^]^ Like other DSB repair factors, TopBP1 is conjugated at DSBs and forms protein condensates, which is a special type of liquid‐liquid phase separation (LLPS).^[^ ^17^, ^35^, ^36^, ^37^, ^38^ ^]^ However, the molecular mechanism by which the LLPS occurs is unclear. Accumulated analyses on other types of nuclear bodies, such as Cajal bodies and nuclear speckles, suggest that RNA species may serve as scaffolds to mediate LLPS and nuclear body formation.^[^ ^39^, ^40^, ^41^, ^42^, ^43^ ^]^ Thus, it is likely that RNA species may mediate DNA damage‐induced foci formation. In fact, several long non‐coding RNAs have been reported to be involved in DSB repair.^[^ ^38^, ^44^, ^45^, ^46^, ^47^, ^48^, ^49^ ^]^ However, none of them serve as scaffolds at DNA lesions to foci formation of DSB repair factors, such as TopBP1.

In addition to DSB repair in somatic cells, TopBP1 also participates in homologous recombination (HR) in germ cells, in which SPO11 induces DSBs for genomic DNA exchange between maternal and paternal chromosomes.^[^ ^50^, ^51^ ^]^ Interestingly, in male germ cells, the sex chromosomes have little homology and thus cannot pair together except pseudo homologous regions close to telomere.^[^ ^51^ ^]^ However, the X and Y chromosomes can still be cut by SPO11 and numerous DNA damage response (DDR) repair factors are loaded onto these two sex chromosomes during the meiotic prophase.^[^ ^51^ ^]^ Thus, DDR factors and these two chromosomes form a unique LLPS named the XY body in male germ cells during meiotic prophase.^[^ ^51^ ^]^ Inside this nuclear body, TopBP1 is at the unsynapsed region of the X and Y chromosomes and co‐localizes with the activated ATR as well as RAD51, a key enzyme for HR repair, indicating that similar to somatic cells, TopBP1 may also mediate ATR activation and HR repair in germ cells.^[^ ^51^, ^52^, ^53^, ^54^ ^]^ Moreover, our recent study demonstrates that pre‐rRNA also localizes at the unsynapsed region, suggesting a possible functional association between TopBP1 and RNA species.^[^ ^55^ ^]^

In this study, we show that the BRCT4‐5 of TopBP1 recognizes pre‐rRNA at DNA lesions, which activates ATR‐dependent signaling pathway and facilitates HR repair. Thus, these findings suggest that pre‐rRNA plays an important role in DSB repair.

## 2 Results

### 2.1 TopBP1 Associates with Pre‐rRNA in Response to DSBs

To examine if any RNA species mediates the DNA damage‐induced foci formation of TopBP1, we pretreated cells with RNase A to remove RNA species, and then stained the cells with anti‐TopBP1 antibody. Interestingly, we found that RNase A treatment erased the foci of TopBP1, suggesting that RNA may be involved in TopBP1 foci formation (Figure S1, Supporting Information).

**Figure 1:**
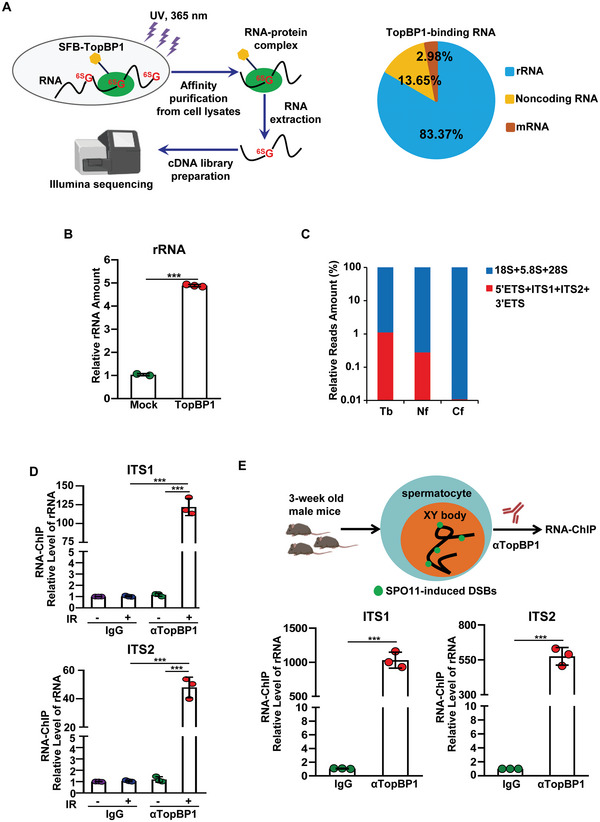
TopBP1 recognizes pre‐rRNA. A) TopBP1 is associated with rRNA. The procedure of PAR‐CLIP and RNA‐Seq from cells stably expressing SFB‐TopBP1 is shown in the left panel. TopBP1‐associated RNA is shown in the right panel. B) TopBP1 specifically recognizes rRNA. Total sequencing amount on 45S rDNA loci from control SFB‐vector (*n* = 2) or SFB‐TopBP1 (*n* = 3) purification are compared. C) Pre‐rRNA is enriched from the purification of TopBP1. The relative RNA sequencing reads amount from TopBP1‐binding (Tb), nucleolar fraction (Nf), or cytoplasmic fraction (Cf) in the regions of 45S pre‐rRNA were compared. D) Endogenous TopBP1 binds to pre‐rRNA in response to IR treatment. Following 10 Gy of IR treatment, the RNA‐ChIP (RIP) assays were performed with control IgG or anti‐TopBP1 antibodies. Pre‐rRNA was analyzed by RT‐qPCR with primers targeting ITS1 and ITS2 regions. E) TopBP1 is associated with pre‐rRNA in male germ cells. Spermatocytes were isolated from 3‐week‐old male mice, followed by RIP with IgG or anti‐TopBP1 antibody (upper panel). RT‐qPCR analysis was applied to measure pre‐rRNA level in spermatocytes (lower panels). Data are represented as mean ± SD as indicated from three independent experiments. Two‐tailed Student's *t‐*test is used to determine statistical significance. ^***^ *p* < 0.001, versus control groups.

To search for the potential RNA species that associates with TopBP1, we established 293T cells stably expressing SFB‐TopBP1 and performed photoactivatable ribonucleoside‐enhanced crosslinking and immunoprecipitation (PAR‐CLIP) (Figure S2A, Supporting Information). Unexpectedly, RNA sequencing results show ribosomal RNA (rRNA) as the major RNA species associated with TopBP1 (Figure **1**A,B). rRNA is transcribed by RNA polymerase I (Pol I) from rDNA loci. Upon transcription, 47S pre‐rRNA is quickly processed at 5′ETS region to generate 45S pre‐rRNA that contains 5′ETS region, 18S region, ITS1, 5.8S region, ITS2, 28S region, and 3′ETS. With extensive processing, 5′ETS, ITS1, ITS2, and 3′ETS are removed, and the remaining 18S, 5.8S, and 28S become mature rRNA.^[^ ^56^, ^57^ ^]^ In the RNA sequencing results, we identified the sequencing reads mainly covering the 18S and 28S regions (Figure 1C and Figure S2B, Supporting Information). In addition, the sequencing reads covering the 5′ transcribed spacers (5′ETS), the internal transcribed spacers 1 (ITS1), 5.8S, the internal transcribed spacers 2 (ITS2), and 3′ external transcribed spacers (3′ETS) were also identified (Figure 1C and Figure S2B, Supporting Information). Compared with RNA sequencing results from total RNA isolated from the cytoplasmic fraction of the cell lysates, the sequencing reads on 5′ETS, ITS1, ITS2, and 3′ETS are remarkably enriched, which is similar to those in the nucleolar fraction (Figure 1C). Thus, these results suggest that pre‐rRNA is enriched from the purification of TopBP1. Since ITS1 and ITS2 only exist in pre‐rRNA, we examined these two regions to validate the results of PAR‐CLIP assays. We performed RT‐qPCR, and found that ectopically expressed SFB‐TopBP1 was associated with pre‐rRNA (Figure S2C, Supporting Information). Next, we examined the endogenous TopBP1 under genotoxic stress condition. We performed RNA‐chromatin immunoprecipitation (RNA‐ChIP) assay using anti‐TopBP1 antibody, and found that endogenous TopBP1 was associated with pre‐rRNA in response to IR treatment as well (Figure 1D, and Figure S3A, B, Supporting Information). RNA‐ChIP results suggest that the interaction between pre‐rRNA and TopBP1 is induced by DSBs. Next, we treated cells expressing SFB‐TopBP1 with 10 Gy of IR and found that SFB‐TopBP1 forms DSB‐induced foci that were not in nucleoli (Figure S3C, Supporting Information), indicating that the SFB‐TopBP1 was associated with pre‐rRNA outside of nucleoli in response to DSBs.

Moreover, TopBP1 localized at the unsynapsed region at the XY body, a major DSB repair hub in male germ cells.^[^ ^51^ ^]^ We isolated spermatocytes from 3‐week‐old male mice, in which a large fraction of spermatocytes is arrested at meiotic prophase with the XY body formation. We examined TopBP1‐associated RNA with RNA‐ChIP. Again, we found that pre‐rRNA was associated with TopBP1 in male germ cells (Figure 1E). Collectively, these results suggest that TopBP1 is associated with pre‐rRNA particularly in response to DSBs.

### 2.2 Pre‐rRNP Colocalizes with TopBP1 at DSBs

**Figure 2:**
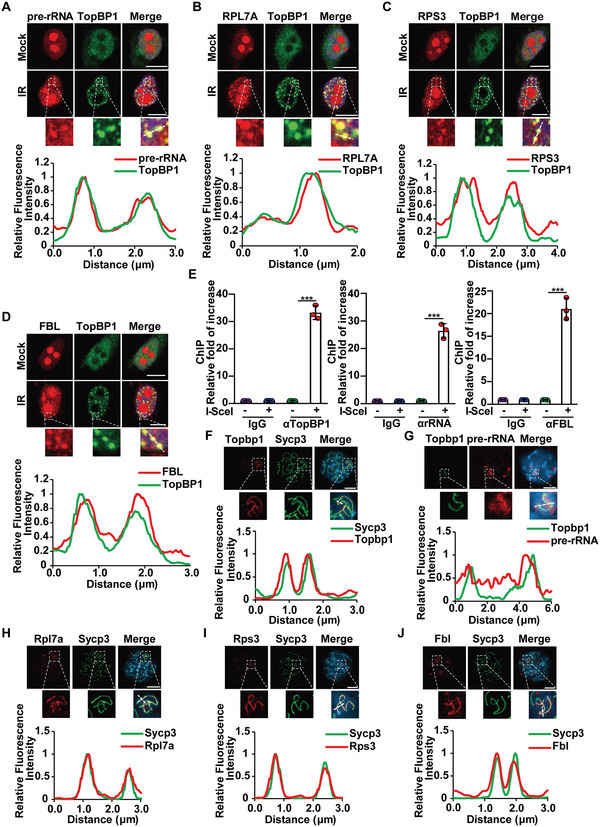
Pre‐rRNPs colocalize with TopBP1 at DSBs. A) Pre‐rRNA colocalizes with TopBP1 at DSBs. Following 10 Gy of IR treatment on HCT116 cells, RNA FISH with pre‐rRNA probes and IF with anti‐TopBP1 antibody were performed. B–D) Pre‐rRNA‐associated proteins colocalize with TopBP1 at DSBs. Following 10 Gy of IR, IF was performed with anti‐TopBP1 and anti‐RPL7A (B), or anti‐RPS3 (C), or anti‐FBL antibodies (D). E) Pre‐rRNPs localize at I‐SceI‐induced DSB. I‐SceI‐mediated DSB was induced by Dox and TA treatment in the HCT116 cells harboring a solo I‐SceI site. Then, ChIP assay with the indicated antibodies was performed. Data are represented as mean ± SD as indicated from three independent experiments. F) TopBP1 localizes at the unsynapsed region of the XY body. Meiotic spread was examined with anti‐TopBP1 antibody. Sycp3 was a surrogate marker of the unsynapsed axis of the XY body in pachytene cells. G) Pre‐rRNA localizes at the unsynapsed region. Pre‐rRNA was examined by RNA FISH with pre‐rRNA probes. H–J) Pre‐rRNA‐associated proteins localize at the unsynapsed region. Meiotic spreads were stained with anti‐Sycp3 and anti‐RPL7A (H), anti‐RPS3 (I), or anti‐FBL antibodies (J). The relative intensity of fluorescence signals is analyzed. Two‐tailed Student's *t*‐test is used to determine statistical significance. ^***^ *p* < 0.001, versus control groups. Scale bars, 10 µm.

In response to IR treatment, TopBP1 is quickly recruited to DSBs and forms foci.^[^ ^17^ ^]^ Since TopBP1 is associated with pre‐rRNA in response to DSBs, we ask if pre‐rRNA relocates to DSBs. We designed specific probes targeting pre‐rRNA and performed RNA FISH. Without induction of DSBs, pre‐rRNA localizes in nucleoli. However, following IR treatment, pre‐rRNA co‐localized with TopBP1 at DSBs and formed foci (Figure **2**A, and Figure S4A,B, Supporting Information). Since pre‐rRNA also associates with protein subunits to form pre‐rRNPs,^[^ ^57^, ^58^, ^59^ ^]^ we also examined and found that RPL7A, RPS3, and FBL localized at DSBs, suggesting that pre‐rRNP is recruited to DSBs (Figure 2B–D and Figure S4C–E, Supporting Information). We subsequently performed co‐immunoprecipitation (co‐IP), and found that these proteins interacted with TopBP1 (Figure S5, Supporting Information). Moreover, these interactions were clearly increased following IR treatment, suggesting that TopBP1 associates with pre‐rRNP in response to DSBs. In addition, these interactions were abrogated following RNase A treatment but not DNase I treatment, indicating that RNA mediates these interactions (Figure S5, Supporting Information).

To validate the results of IR‐induced foci (IRIF) of TopBP1, an I‐SceI site is knocked into the X chromosome of HCT116 cells. With inducible I‐SceI, a solo DSB is generated (Figure S6, Supporting Information).^[^ ^60^ ^]^ We performed ChIP and qPCR assays and found that rRNA and FBL were enriched at the flanks of I‐SceI‐induced DSB (Figure 2E). Taken together, these results suggest that pre‐rRNP co‐localizes with TopBP1 at DSBs.

In addition to DSBs in somatic cells, we examined the localization of pre‐rRNA in the XY body. We used probes specifically targeting pre‐rRNA for RNA FISH, and found that both TopBP1 and pre‐rRNA localize at the unsynapsed region of the XY body (Figure 2F,G). Next, we also examined RPL7A, RPS3, and FBL. All these pre‐rRNA‐associated proteins localized at the unsynapsed region of the XY body (Figure 2H–J), further indicating that pre‐rRNP associates with TopBP1 at DSBs.

### 2.3 Pre‐rRNA Mediates the Recruitment of TopBP1 to DNA Lesions

**Figure 3:**
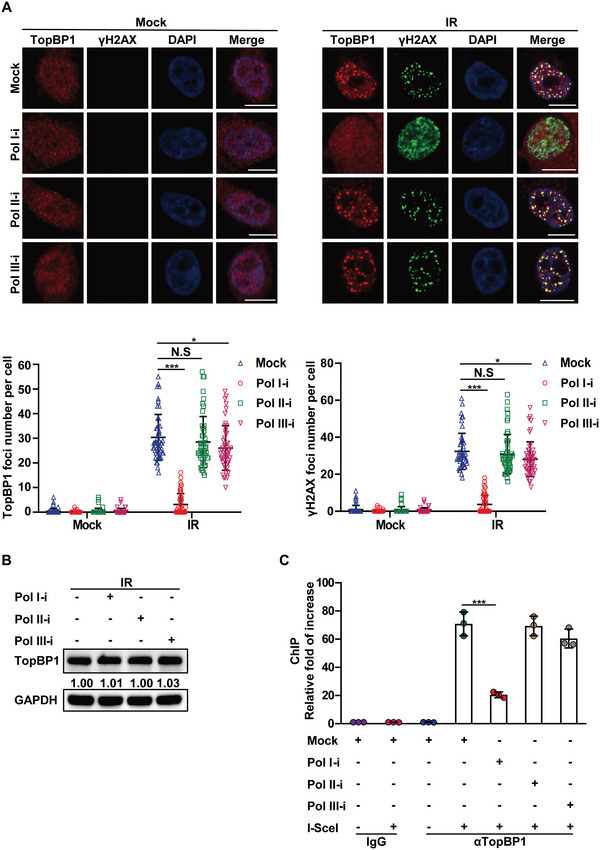
Pol inhibitor treatment suppresses the recruitment of TopBP1 to DSBs. A) RNA Pol I inhibitor treatment represses the IRIF of TopBP1. Following Pol I, Pol II, or Pol III inhibitor treatment, HCT116 cells were treated with or without 10 Gy of IR. IRIF of TopBP1 and γH2AX was examined by IF with anti‐TopBP1 and anti‐γH2AX antibodies respectively (upper panels). The foci number in each cell was counted (lower panels). The bars represent the mean values ± SD (*n*  =  50 from three independent experiments, per group). B) The protein level of TopBP1 is not affected by Pol I inhibitor treatment. HCT116 cells were treated with Pol I, Pol II, or Pol III inhibitors. The protein level of TopBP1 was examined by Western blot. GAPDH was used as protein loading controls. C) Pol I inhibitor treatment suppresses the recruitment of TopBP1 to I‐SceI‐induced DSB. Following Pol I, Pol II, or Pol III inhibitor treatment, the accumulation of TopBP1 at the I‐SceI‐induced DSB was examined by ChIP assay. Data are represented as mean ± SD as indicated from three independent experiments. Two‐tailed Student's *t*‐test is used to determine statistical significance. ^*^ *p* < 0.05; ^***^ *p* < 0.001; N.S, not significant, versus control groups. Scale bars, 10 µm.

To examine the functional interactions between pre‐rRNA and TopBP1 at DSBs, we used siRNA to knockdown TopBP1. However, lacking TopBP1 did not affect the loading of pre‐rRNA to DSBs (Figure S7, Supporting Information). Next, when we transiently treated cells with Pol I inhibitor (BMH‐21) to ablate the pre‐rRNA biogenesis but not affect protein translation (Figure S8, Supporting Information), we found that RNA Pol I inhibitor treatment suppressed the pre‐rRNA foci at DSBs (Figure S9, Supporting Information). Interestingly, Pol I inhibitor treatment clearly suppressed the IRIF of TopBP1. In sharp contrast, Pol II inhibitor (α‐Amanitin) treatment did not affect the IRIF of TopBP1 (Figure **3**A). We also treated cells with Pol III inhibitor (ML‐60218). Since Pol III mediates 5S rRNA transcription, we found that Pol III inhibitor slightly suppressed TopBP1 foci formation (Figure 3A). The impaired foci formation was not caused by downregulation of TopBP1 (Figure 3B). We also examined the role of pre‐rRNA in the recruitment of TopBP1 to the I‐SceI‐induced DSB. Consistently, ChIP assay results show that Pol I inhibitor treatment largely suppressed the recruitment of TopBP1 to the DSB (Figure 3C).

In response to DSBs, H2AX is phosphorylated by ATM.^[^ ^61^ ^]^ Phosphorylated H2AX, also known as γH2AX, provides a platform to mediate the loading of DSB repair factors at DSBs.^[^ ^62^, ^63^ ^]^ Next, we examined and found that the foci of pre‐rRNA were suppressed in H2AX‐deficient cells, suggesting that γH2AX plays a crucial role in the foci formation of pre‐rRNA at DSBs (Figure S10A, Supporting Information). Moreover, when we treated cells with ATM inhibitors, we found that both the foci formation of γH2AX and pre‐rRNA was impaired (Figure S10B,C, Supporting Information), suggesting that ATM‐γH2AX axis mediates the recruitment of pre‐rRNA to DSBs. Interestingly, when we treated cells with RNase A to digest RNA species at DSBs, we found that the foci of γH2AX were impaired as well (Figure S1, Supporting Information). These results suggest that a functional interaction between γH2AX‐MDC1 and pre‐rRNA mediates DSB‐induced foci formation (Figure S10D, Supporting Information).

### 2.4 The BRCT4‐5 Domain of TopBP1 Recognizes Pre‐rRNA

**Figure 4:**
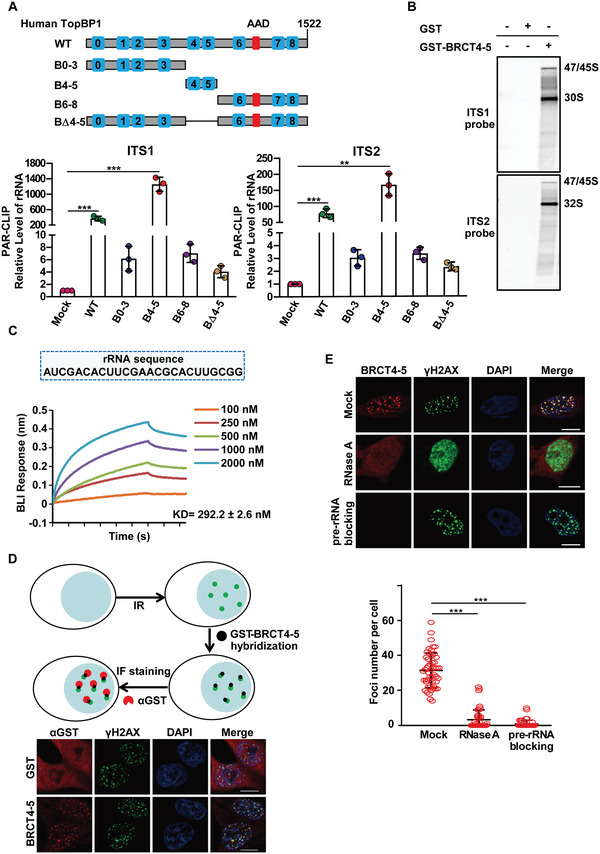
TopBP1 BRCT4‐5 domain recognizes pre‐rRNA. A) PAR‐CLIP assays of full‐length and mutant TopBP1. Schematic showing the conserved domains of TopBP1 as well as the deletion mutants of TopBP1 (upper panel). AAD: ATR‐activating domain; nine BRCTs are shown from 0 to 8. HEK293T cells expressing empty vector (mock), SFB‐tagged full‐length or deletion mutants of TopBP1 were subjected to the PAR‐CLIP assays. RT‐qPCR analysis was applied to measure the level of pre‐rRNA using probes targeting ITS1 or ITS2 regions (lower panels). Data are represented as mean ± SD as indicated from three independent experiments. B) TopBP1 BRCT4‐5 domain associates with pre‐rRNA. Total RNA isolated from cells was incubated with GST or GST‐TopBP1‐BRCT4‐5, followed by pull‐down assay. The RNA species isolated by pull‐down assay were analyzed by Northern blot. C) The BRCT4‐5 domain of TopBP1 directly interacts with pre‐rRNA. The binding affinity between the recombinant TopBP1 BRCT4‐5 domain and 25 nt biotin‐labeled pre‐rRNA was analyzed by BLI. D) The BRCT4‐5 domain of TopBP1 recognizes IRIF. The recombinant GST‐TopBP1‐BRCT4‐5 was incubated with IR (10 Gy) treated HeLa cells. The hybridized protein was examined by anti‐GST antibodies. E) The IRIF of recombinant GST‐TopBP1‐BRCT4‐5 is mediated by RNA. IR‐treated HeLa cells were further treated with RNase A or blocked with excessive pre‐rRNA prior to GST‐TopBP1‐BRCT4‐5 hybridization (upper panels). The foci number was counted (lower panel). The bars represent the mean values ± SD (*n*  =  50 from three independent experiments, per group). Two‐tailed Student's *t*‐test is used to determine statistical significance. ^**^ *p* < 0.01; ^***^ *p* < 0.001, versus control groups. Scale bars, 10 µm.

Next, we sought to further characterize the interaction between TopBP1 and pre‐rRNA. TopBP1 contains nine BRCT domains. In order to map the interaction region, we generated a series of deletion mutants of TopBP1 (Figure **4**A). Using PAR‐CLIP assays, we found that the BRCT4 and BRCT5 domains were associated with pre‐rRNA (Figure 4A and Figure S11A, Supporting Information). Structure analyses show that these two BRCT domains stack on each other forming a heterodimer.^[^ ^64^ ^]^ A phosphate‐binding pocket exists in the BRCT5, whereas the BRCT4 facilitates the folding of the BRCT5. To examine the function of this phosphate‐binding pocket, we generated the K704A mutation to abolish the phosphate‐binding pocket, and found that this mutation largely abrogated the interaction between TopBP1 and pre‐rRNA (Figure S11B,C, Supporting Information).

To further validate the interaction between the BRCT4‐5 and pre‐rRNA, we generated the recombinant BRCT4‐5 of TopBP1 and incubated it with pre‐rRNA. Using in vitro pull‐down assay and RT‐qPCR, we found that the BRCT4‐5 bound to pre‐rRNA in vitro (Figure S11D, Supporting Information). Next, we incubated TopBP1 BRCT4‐5 with total RNA isolated from cell lysates, and performed Northern blot. Again, the BRCT4‐5 was able to enrich pre‐rRNA from the cell lysates (Figure 4B). Moreover, we measured the affinity between the BRCT4‐5 and 25 nt biotin‐labeled pre‐rRNA oligos using Bio‐layer interferometry (BLI). The sequence of RNA oligos is derived from the most frequently found RNA sequencing reads from the PAR‐CLIP assays. The KD was around 300 nm, demonstrating that the BRCT4‐5 of TopBP1 directly interacts with pre‐rRNA in vitro (Figure 4C). In addition, we shortened the RNA length and found that the BRCT4‐5 domain was even able to bind to 6 nt or 3 nt RNA oligos (Figure S12, Supporting Information).

Next, we ask if the BRCT4‐5 recognizes the pre‐rRNA at DSBs. We performed protein hybridization assays by incubating the BRCT4‐5 with IR‐irradiated cells (Figure 4D). The BRCT4‐5 was able to hybridize to DSBs, suggesting that the BRCT4‐5 recognizes its functional partner(s) at DSBs (Figure 4D). However, with the treatment of RNase A, the foci of the BRCT4‐5 were suppressed. Moreover, in the BRCT4‐5 hybridization assay, we preincubated the BRCT4‐5 with pre‐rRNA to mask the phosphate binding pocket in the BRCT5. We found that excessive pre‐rRNA blocked the hybridization foci of the BRCT4‐5, suggesting that it is pre‐rRNA to mediate the recruitment of the BRCT4‐5 to DSBs (Figure 4E). In addition to DSBs in somatic cells, protein hybridization assays show that TopBP1 BRCT4‐5 recognized the unsynapsed regions of the XY body in male germ cells, and such localization was suppressed by the RNase A treatment or pre‐rRNA blocking (Figure S13, Supporting Information). Collectively, these results demonstrate that TopBP1 BRCT4‐5 domain recognizes pre‐rRNA at DSBs.

### 2.5 TopBP1 Forms Phase Separation with Pre‐rRNA

**Figure 5:**
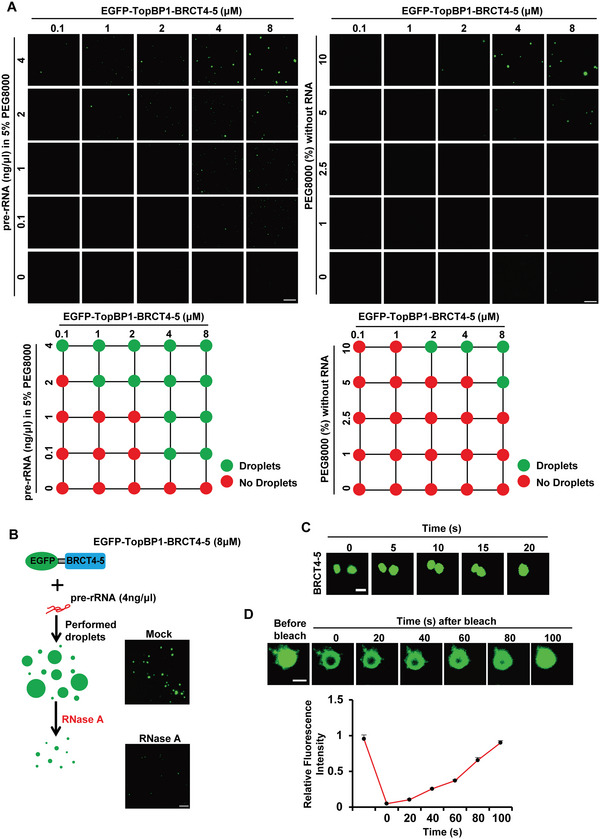
TopBP1 and pre‐rRNA form liquid‐liquid phase separation in vitro. A) Droplet formation of the TopBP1‐pre‐rRNA complex. Recombinant TopBP1‐BRCT4‐5 was incubated with pre‐rRNA in the presence of 5% PEG8000 (upper left panels) or with indicated concentrations of PEG8000 (upper right panels). LLPS was observed by fluorescence microscopy. Corresponding square networks depict summaries of TopBP1‐BRCT4‐5 droplet formation (lower panels). B) Droplet formation of the TopBP1‐pre‐rRNA complex is suppressed by RNase A treatment. Recombinant TopBP1‐BRCT4‐5 was incubated with pre‐rRNA, followed by 1 mg mL^−1^ RNase A treatment. C) Microscopy images of liquid droplets fusion of the TopBP1‐pre‐rRNA complex at indicated time points. D) Representative micrographs of TopBP1‐BRCT4‐5 liquid droplets before and after photobleaching (upper panels). Fluorescence intensity of FRAP was quantified (lower panel). Image bar: 1 µm.

The foci formation of TopBP1 is a unique type of protein that condensates in the nucleus.^[^ ^17^, ^35^, ^36^, ^37^, ^38^ ^]^ To explore the underlying mechanism of the foci formation, we examine LLPS formation of the TopBP1 and pre‐rRNA complex. We generated recombinant GFP‐BRCT4‐5, and incubated the protein with pre‐rRNA. Interestingly, in the presence of pre‐rRNA, GFP‐BRCT4‐5 formed liquid droplets in a concentration‐dependent manner (Figure **5**A). RNase A treatment decreased the liquid droplets, suggesting that pre‐rRNA plays a key role to mediate the liquid droplets (Figure 5B). Usually, during liquid droplet formation, tiny droplets are fused into larger ones that can be detected under light microscopy.^[^ ^65^ ^]^ Here, we observed this dynamic liquid droplet fusion process (Figure 5C), further demonstrating the liquid droplet formation in the presence of the BRCT4‐5 and pre‐rRNA. Moreover, one important characteristic of liquid droplets is that dynamic exchange of molecules occurs between liquid droplets and the surrounding solution.^[^ ^65^, ^66^ ^]^ Here, we performed fluorescence redistribution after photo‐bleaching (FRAP) assays to validate this process. We found that photo‐bleaching‐induced loss of fluorescence was quickly recovered (Figure 5D). Collectively, these results suggest that pre‐rRNA promotes protein condensates of TopBP1 in vitro, which may be the underlying molecular mechanism of TopBP1 foci formation in cells.

### 2.6 TopBP1‐Mediated ATR/CHK1 Signaling Pathway is Regulated by Pre‐rRNA

**Figure 6:**
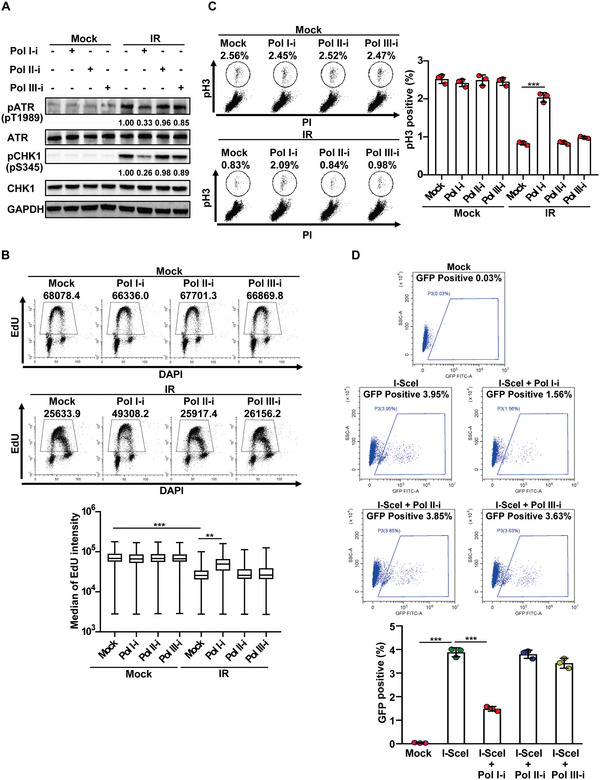
Pre‐ribosomal RNA mediates DSB response. A) Pol I inhibitor treatment suppresses ATR activation. HCT116 cells were treated with RNA Pol I, Pol II, or Pol III inhibitor for 2 h, followed by 10 Gy of IR. Then, Immunoblotting was performed with the indicated antibodies. Numbers below lanes (top) indicate densitometry of the protein presented relative to ATR or CHK1 expression in the same lane. B) Pol I inhibitor treatment impairs intra‐S phase checkpoint. HCT116 cells were pulse labeled with 10 µm EdU for 30 minutes after IR (10 Gy). Labeled S phase cells were examined by flow cytometry (upper panels). Median EdU intensity was plotted (lower panel). C) Pol I inhibitor treatment impairs G2/M checkpoint. HCT116 cells were treated with or without IR (2 Gy). The G2/M checkpoint was examined by flow cytometry with anti‐phospho‐histone H3 (pH3) antibodies (left panels). The percentage of pH3‐positive cells in C was statistically analyzed (right panel). D) Pol I inhibitor treatment suppresses HR repair. Following Pol I, Pol II, or Pol III inhibitor treatment, DR‐GFP‐U2OS cells were used to measure the HR efficiency, and GFP positive population was examined by flow cytometry (upper panels). The percentage of GFP‐positive cells was statistically analyzed (lower panel). Data are represented as mean ± SD as indicated by three independent experiments. Two‐tailed Student's *t*‐test is used to determine statistical significance. ^**^ *p* < 0.01; ^***^ *p* < 0.001, versus control groups.

In response to DSBs, TopBP1 is required for the activation of ATR as well as for HR repair.^[^ ^67^ ^]^ Since pre‐rRNA is a partner of TopBP1, we examine if pre‐rRNA mediates the functions of TopBP1 in DNA damage response. Following DSBs, ATR autophosphorylates itself at Thr1989, a surrogate marker of the ATR activation.^[^ ^68^ ^]^ With Pol I inhibitor treatment, we found that the level of pT1989 was remarkably suppressed (Figure **6**A). Moreover, activated ATR phosphorylates CHK1 at Ser345 for signal transduction.^[^ ^11^, ^13^ ^]^ Again, Pol I inhibitor treatment suppressed pS345 of CHK1 (Figure 6A). Finally, CHK1 is required for the activation of the S phase checkpoint and G2/M checkpoint to arrest the cell cycle.^[^ ^13^, ^14^ ^]^ With Pol I inhibitor treatment, these two cell cycle checkpoints were suppressed (Figure 6B,C). In contrast, Pol II inhibitor treatment impaired neither the activation of the ATR‐dependent signaling pathway nor the DNA damage‐induced cell cycle checkpoint (Figure 6A–C).

TopBP1 is also known to facilitate HR repair by mediating the recruitment of RAD51 to DSBs.^[^ ^69^, ^70^ ^]^ To investigate the role of pre‐rRNA in TopBP1‐mediated HR repair, we used an established GFP reporter system, and confirmed that TopBP1 played an important role in HR repair (Figure S14, Supporting Information). Moreover, we examined and found that the loss of TopBP1 suppressed foci of RAD51 (Figure S15, Supporting Information), which is in agreement with earlier studies.^[^ ^69^ ^]^ Similar to the loss of TopBP1, we found that Pol I inhibitor treatment, but not Pol II inhibitor repressed HR (Figure 6D). Moreover, the effect of Pol I inhibitor treatment did not further reduce the HR efficacy in cells lacking TopBP1, suggesting the possible epistasis of pre‐rRNA and TopBP1 in the regulation of HR repair (Figure S14, Supporting Information). Collectively, these results suggest that pre‐rRNA mediates the functions of TopBP1 during DNA damage response (Figure S16, Supporting Information).

## 3 Discussion

In this study, we demonstrate pre‐rRNA as a functional partner of TopBP1 in DSB repair. The TopBP1 BRCT4‐5 recognizes pre‐rRNA for DNA damage‐induced foci formation. Numerous studies have shown that RNA species is a scaffold for nuclear body formation because RNA mediates LLPS of many protein complexes in the nucleus.^[^ ^39^, ^40^, ^41^, ^42^, ^43^ ^]^ DNA damage‐induced foci formation is a unique type of LLPS in the nucleus. However, pre‐rRNA has not been identified in the foci of TopBP1 until this study. rRNA is often considered as a source of RNA contamination. Thus, the very first step of RNA sequencing analysis is to remove rRNA (aka “riboclear”).^[^ ^71^, ^72^ ^]^ Here, we demonstrate that pre‐rRNA is not RNA contamination, but localizes at DSBs. In particular, it localizes not only at genotoxic stress‐induced DSBs in somatic cells, but also at physiologically relevant DSBs in the XY body of male germ cells. Moreover, we found that only RNase A treatment but not RNase H treatment could impair the interactions between TopBP1 and pre‐rRNA (Figure S1, Supporting Information). Thus, it excludes the possibility of R‐loops at rDNA loci mediating the foci formation of TopBP1. Instead, pre‐rRNA may act as a scaffold for the recruitment of TopBP1 to DSBs.

The BRCT domain is originally known as phoshpho‐Ser binding motif.^[^ ^19^, ^20^, ^21^, ^22^ ^]^ Here, we found that the BRCT4‐5 of TopBP1 is able to directly bind to pre‐rRNA with high affinity. Since pre‐rRNA has a lot of phosphate moieties on its backbone, it is likely that the BRCT4‐5 may recognize phosphate moieties in pre‐rRNA. Since the interactions between protein and RNA are always multivalent,^[^ ^73^, ^74^ ^]^ it is likely that the BRCT5 recognizes other groups on pre‐rRNA to selectively interact with pre‐rRNA. The detailed interaction mode should be further explored with structural analysis. In our study, we found that the BRCT4‐5 recognized no more than three‐nucleotide oligo, suggesting that other pre‐rRNA binding motifs of TopBP1 facilitate the selective binding to pre‐rRNA. In addition to the BRCT4‐5, TopBP1 has other eight BRCT domains, and several functional partners have been identified. Thus, it is possible that other BRCT domains of TopBP1 or its associated proteins may also bind to pre‐rRNA.

The results from the PAR‐CLIP assays show that most TopBP1‐associated RNA sequencing reads are in the 18S and 28S regions. Consistently, the 45S pre‐rRNA is extremely unstable,^[^ ^75^, ^76^, ^77^ ^]^ and majorities of pre‐rRNA species are highly processed pre‐rRNA.^[^ ^78^, ^79^ ^]^ Thus, it is possible that TopBP1 recognizes motifs in these regions of pre‐rRNA. However, considering that the 28S region is the largest region in pre‐rRNA, it is very easy to identify this region by RNA sequencing. Even when we purify and analyze pre‐rRNA isolated from nucleoli fractions, the most abundant peaks always exist in the 18S and 28S regions (Figure 1C and Figure S2B, Supporting Information). We postulate that these two regions are well‐folded, thus more stable than other regions during RNA manipulation in vitro. Alternatively, the GC‐rich region‐caused secondary in pre‐rRNA may cause system bias during RNA sequencing. At the current stage, it is very difficult to identify the interaction motifs on pre‐rRNA. Perhaps, future motif scanning on the complicated and folded pre‐rRNA may reveal the binding motifs. In addition, in the PAR‐CLIP assays, we also found a number much less abundant RNA enriched from the purification of TopBP1 (GSE213073). It is possible that other RNA may associate with TopBP1. In particular, at the current stage, we do not know if TopBP1 recognizes specific motifs on RNA. It is possible that other RNA may function together with TopBP1 in different stages of DSB repair.

Under normal physiological conditions, pre‐rRNA is synthesized and processed in nucleolus.^[^ ^78^, ^79^ ^]^ After extensive modifications, pre‐rRNA associates with other protein subunits forming pre‐rRNP that is quickly shipped to cytoplasm. Here, we observed IR‐induced association between TopBP1 and pre‐rRNA. It is possible that during DSB response, pre‐rRNA is released from nucleoli, recruited to DSBs, and recognized by TopBP1. Our previous study on the XY body indicates that MDC1, the functional partner of γH2AX, is required for loading pre‐rRNA to DSBs.^[^ ^55^ ^]^ Consistently, here we found that the ATM‐ γH2AX axis determined the foci formation of pre‐rRNA. Interestingly, RNase A treatment to remove pre‐rRNA at DSBs also impaired the foci of γH2AX, suggesting that both pre‐rRNA and γH2AX are scaffolds to maintain IR‐induced foci (Figure S10D, Supporting Information).

Previous studies have revealed the biological functions of TopBP1 in DNA damage response.^[^ ^7^, ^17^, ^18^ ^]^ Here, we reveal that pre‐rRNA mediates the foci formation of TopPB1 at DSBs, and ATR‐governed signaling transduction, cell cycle checkpoint activationm and HR repair. Pre‐rRNA is considered as a precursor of rRNA, but here we show that pre‐rRNA functions in DSBs response. The detailed molecular mechanism by which ATR is activated remains elusive. It has been shown that the AAD motif of TopBP1 interacts with ATR and ATRIP complex for the activation of ATR. It is likely that pre‐rRNA mediates the recruitment of TopBP1 to DSBs, where TopBP1 activates ATR. We also understand that other possible mechanisms may exist to explain the activation of ATR. For example, similar to TopBP1, the recruitment of pRPA and Rad9 to DNA lesions was also suppressed by pol I inhibitor treatment (Figure S17, Supporting Information). Thus, it is possible that ATR activation is regulated by pre‐rRNA through multiple layers. In addition to DSBs, ATR‐dependent pathway is activated during replication stress, which is also dependent on the RPA complex and 9‐1‐1 complex, but not γH2AX.^[^ ^9^, ^80^, ^81^ ^]^ Currently, we do not know if pre‐rRNA is involved in these cellular processes. Nevertheless, here we provide the first evidence that pre‐rRNA associates with ATR activation in response to DSBs. Of note, a recent study shows that TopBP1 condensation stimulates ATR activation.^[^ ^35^ ^]^ Interestingly, this process is associated with NOL11, a nucleolar protein.^[^ ^35^ ^]^ Thus, it is possible that pre‐rRNA‐associated proteins may also participate in ATR activation.

In addition to regulating global ATR activation, TopBP1 also specifically mediates ATR activation at lesions on the rDNA region.^[^ ^82^, ^83^, ^84^ ^]^ Several studies have shown that TopBP1 is associated with Treacle for ATR activation upon replication stress at rDNA loci.^[^ ^82^, ^83^, ^84^ ^]^ This process results in the suppression of pre‐rRNA synthesis.^[^ ^85^, ^86^, ^87^ ^]^ Here, we found that pre‐rRNA may facilitate TopBP1‐dependent DNA damage response. It is possible that pre‐rRNA may also interact with TopBP1 in nucleolus for the replication stress response at rDNA loci.

Our study shows that the interaction between pre‐rRNA and TopBP1 is induced by DSBs. Pre‐rRNA is synthesized by RNA Pol I and processed by numerous enzymes in nucleoli.^[^ ^78^, ^79^ ^]^ Once it is processed, pre‐rRNA associates with other chaperones and quickly translocates from nucleoli to cytoplasm.^[^ ^78^, ^79^ ^]^ Since IR treatment induces ribosomal biogenesis stress, transcription of pre‐rRNA is transiently suppressed, transcription machinery and processing factors relocate to the edge of nucleoli or are released from the nucleoli.^[^ ^88^, ^89^, ^90^ ^]^ In this study, we found that pre‐rRNA also relocated to DSBs. Thus, during the DSBs response, pre‐rRNA is released from nucleoli, recruited to DSBs, and recognized by TopBP1. Alternatively, it is possible that exportation of processed pre‐rRNP from nucleoli to cytoplasm may be affected when DSBs occur, which induces the relocation of pre‐rRNA to DSBs. Moreover, it is possible that other DSB repair factors may also recognize pre‐rRNA. In particular, the BRCT domain has been identified in several DNA damage repair factors. We anticipate that more pre‐rRNA‐mediated interactions will be uncovered during DNA damage repair in the near future.

## 4 Experimental Section

### Cell Culture and Animals

HEK293T, HCT116, HeLa, DR‐GFP‐U2OS, WT MEFs, and *H2ax^−/−^* MEFs were cultured in DMEM supplemented with 10% fetal bovine serum, 1% penicillin, and 1% streptomycin. Wildtype male mice were obtained from the Westlake University Animal Center. All animal experiments were performed by strictly adhering to the Guide for the Care and Use of Laboratory Animals. The protocols for animal experiments were approved by the Westlake University Animal Care and Use Committee (approval number 20‐029‐YXC and 20‐037‐YXC).

### Plasmid Constructs and siRNAs

The cDNA encoding full‐length and deletion mutants of human TopBP1 was inserted into SFB vector for the ectopic expression in mammalian cells. To generate GST‐TopBP1‐BRCT4‐5, the cDNA encoding TopBP1‐BRCT4‐5 was inserted into the pGEX‐4T‐1 vector. The cDNA was amplified with the following primers:

EcoRI‐TopBP1‐BRCT4‐5‐Forward: cggGAATTCACTGAAGAAGGCTTATTTAGCCAAAAG

XhoI‐TopBP1‐BRCT4‐5‐Reverse: cggCTCGAGTTACAAACTTCGTTCTTCTTTAGTTG

For GFP‐TopBP1‐BRCT4‐5, the pEGFP‐C1‐TopBP1‐BRCT4‐5 construct was generated. Then, the cDNA encoding GFP‐TopBP1‐BRCT4‐5 was subcloned into pGEX‐4T‐1 vector with the following primers:

XhoI‐TopBP1‐BRCT4‐5‐Forward: cggCTCGAGctACTGAAGAAGGCTTATTTAGCCAAAAG

EcoRI‐TopBP1‐BRCT4‐5‐Reverse: cggcggGAATTCTTACAAACTTCGTTCTTCTTTAGTTG

recombinant cloning primer Forward:

TTCCGCGTGGATCCCCGGAATTCATGGTGAGCAAGGGCGAGGAGCTG

recombinant cloning primer Reverse:

GATCGTCAGTCAGTCACGATGCGGCCGCTTACAAACTTCGTTCTTCTTTAG

siRNA targeting human TopBP1 was chemically synthesized (Sangon Biotech):

sense: GCGUAAGUGAAUCAAUAUGUATT;

antisense:UACAUAUUGAUUCACUUACGCTT.

siRNA transfection was performed using Lipofectamine RNAiMAX (Invitrogen) according to the manufacturer's protocol.

### Antibodies

Detailed information of antibodies used in this study: anti‐Flag (Sigma, Rabbit, F7425), anti‐TopBP1 (Santa Cruz, Mouse, sc‐271043; BETHYL, Rabbit, A300‐111A), anti‐γH2AX (Novus, Rabbit, NB100‐384; CST, Rabbit, 9718), anti‐SCP3 (Santa Cruz, Mouse, sc‐74569; Novus, Rabbit, NB300‐232), anti‐rRNA (Novus, Mouse, NB100‐662), anti‐RPL7A (ABclonal, Rabbit, A13713), anti‐RPS3 (ABclonal, Rabbit, A2533), anti‐FBL (CST, Rabbit, 2639S; Santa cruz, Mouse, sc‐166001), IgG (Rabbit, CST, 2729), IgG (Mouse, CST, 3420), anti‐ATR (BETHYL, Rabbit, A300‐137A), anti‐Chk1 (Novus, Rabbit, NB100‐464), anti‐pATR (T1989) (GeneTex, Rabbit, GTX128145), anti‐pChk1 (Ser345) (CST, Rabbit, 2348), anti‐GAPDH (CST, Mouse, 97 166), anti‐pH3 (Ser10) (CST, Rabbit, 9701), anti‐Puromycin (Millipore, Mouse, MABE343), anti‐GAPDH (HUABIO, Rabbit, ET1601‐4), anti‐Histone H3 (CST, Rabbit, 4499S), anti‐ATM (GeneTex, Mouse, GTX70107), anti‐pATM (S1981) (ROCKLAND, Mouse, 200‐301‐400), anti‐pRPA (Novus, Rabbit, NBP1‐23017), anti‐Rad9 (Santa Cruz, Mouse, sc‐74464), anti‐RAD51 (abcam, Rabbit, ab63801).

### PAR‐CLIP, RNA Sequencing, and Data Analysis

HEK293T cells stably expressing SFB empty vector or SFB tagged TopBP1 were incubated with DMEM in 37 °C, 5% CO_2_ incubator. 6SG (Sigma) (final concentration, 0.1 mm) was added to the cell culture medium 6 h prior IR. Eight hours after 10 Gy of IR, the cells were placed in a tray filled with ice to keep cells cold and cross‐linked with 0.4 J cm^−2^ total energy of 365 nm UV. Subsequently, for the isolation of nucleolar fraction and cytoplasmic fraction assay, the cells were washed with ice‐cold PBS. Then, the cells were resuspended in a hypotonic buffer (10 mm HEPES pH 7.9, 10 mm KCl, 1.5 mm MgCl_2_, 0.5 mm DTT) in the presence of protease inhibitor cocktail (Roche), phosphatase inhibitor cocktail (Roche), and RNase inhibitor, and then incubated on ice for 5 min. The cells were then ruptured using precooled Dounce homogenizer and the tight pestle (VWR Cat. 62400–595). The homogenized cells were centrifuged at 1000 rpm for 5 min at 4 °C. The supernatant was collected as cytoplasmic fraction. Resuspend the pellet with buffer S1 (0.25 m sucrose, 10 mm MgCl_2_). Layer the resuspended pellet on top of buffer S2 (0.35 m sucrose, 0.5 mm MgCl_2_). Centrifuge at 2500 rpm for 5 min at 4 °C to further purify nuclei. The pellet was resuspended in buffer S2 and sonicated on ice. The sonicated sample was then layered on top of buffer S3 (0.88 m sucrose, 0.5 mm MgCl_2_) and centrifuged at 3500 rpm for 10 min at 4 °C to purify nucleoli. The nucleolar pellet was resuspended in buffer S2 and centrifuged at 2500 rpm for 5 min at 4°C. The pellet that contained highly purified nucleoli was resuspended in buffer S2 as a nucleolar fraction. RNAs from nucleolar fraction and cytoplasmic fraction were extracted by TRIzol reagent (Life Technologies) according to the manufacturer's protocol and then analyzed by sequencing of RNA. For TopBP1‐binding RNA using PAR‐CLIP assay, the cells were lysed in NETN300 (50 mm Tris‐HCl, pH 7.5, 300 mm NaCl, 2 mm EDTA, 1% NP40) in the presence of protease inhibitor cocktail (Roche), phosphatase inhibitor cocktail (Roche), RNase inhibitor and VRC, diluted with NETN0. The cell lysates were incubated with High Capacity Streptavidin Resin for 6 h at 4 °C. The beads were washed three times for 1 h with NETN300. After eluting two times for 1 h with biotin at 4 °C, the elution was incubated with anti‐Flag beads (Sigma) for 6 h at 4 °C. The beads were washed three times for 1 h with NETN300. Then, the protein‐associated RNA was isolated from the beads using proteinase K digestion and precipitated with phenol–chloroform. After ethanol‐precipitated, the RNA was resuspended with nuclease‐free water. Next, sequencing of RNA isolated by PAR‐CLIP was performed by LC‐Bio Technology CO., Ltd., Hangzhou, Zhejiang, China.

The raw fastq files were analyzed using FastQC (v0.11.9). Sequencing adaptors and low‐quality (base quality score<20) reads were trimmed by trim galore (v0.6.6). Reads shorter than 20 nt were discarded. The trimmed reads were aligned to the 45S rDNA locus by STAR (v2.7.8a). The 45S rDNA annotation file was transformed into GTF format using gffread (v0.12.1). The expression levels of 18S, 5.8S, 28S rRNA, ITS1, ITS2, 5′ETS, and 3′ETS were quantified by FeatureCounts (v2.0.1). In addition, the reads that were not mapped to 45S rDNA were aligned to the human GRCh38 CDS and ncRNA (ENSEMBL, release‐105) FASTA sequences by STAR. Total RNA was calculated including reads aligned to the 45S DNA, CDS, or ncRNA. The normalized count (counts per million‐CPM) in 18S rRNA, 28S rRNA, ITS1, ITS2, and 5′ETS regions were quantified in each 200 bp bin. 3′ETS region was quantified in each 100 bp bin and the normalized counts in 5.8S rRNA region were quantified in each 10 bp bin.

### RNA‐ChIP (RIP) Assay

Cells were fixed in 1% formaldehyde for 10 min at room temperature, then quench cross‐linking with 0.125 m glycine for 5 min. Cells were washed twice with cold PBS and collected. Lysate the cells with 10 V of RIP buffer (100 mm KCl, 5 mm MgCl_2_, 10 mm Hepes pH 7.5, 0.5% NP‐40, 1 mm DTT, 2 mm VRC, 100 U mL^−1^ RNase inhibitor, protease inhibitor cocktail). The cells were homogenized by sonication to an average fragment size of 500–1000 nucleotides and then centrifuged at 15 000 rpm for 30 min at 4 °C to remove the insoluble materials. Next, lysates were immunoprecipitated with the indicated primary antibodies following the ChIP protocol. Then, lysates were incubated with ChIP Grade Protein A/G Plus Agarose (Thermo Fisher Scientific) in cold room. After washing the beads, cross‐links were reversed following the ChIP protocol. Then, RNA was precipitated with phenol–chloroform and treated extensively with DNase I. After ethanol‐precipitated, the RNA was resuspended with nuclease‐free water. Then, cDNA was synthesized using the SuperScript III First‐Strand Synthesis System for RT‐PCR kit with random primers (Invitrogen). RT‐qPCR was performed in triplicate using SYBR Green Master Mix on CFX connect Real‐Time PCR Detection System (Biorad). Sequences of the primers:

hITS1‐qPCR‐S, CGAGAGCCGGAGAACTCGG;

hITS1‐qPCR‐AS, GCCGACACCCACGTCGTC;

hITS2‐qPCR‐S, CGGGCCCTGCGTGGTCAC;

hITS2‐qPCR‐AS, GGAGGAACCCGGACCGCAG;

mITS1‐qPCR‐S, GGCTCTCCCTCAGACTCCAT;

mITS1‐qPCR‐AS, GGAAAAACACGGGAACGACA;

mITS2‐qPCR‐S, GTCTCCCGAAGTTCAGACGTG;

mITS2‐qPCR‐AS, GGACCGAGAAAGACTGGTGAG.

### I‐SceI‐Mediated DSBs System and Chromatin Immunoprecipitation (ChIP)

HCT116 cells that have a solo I‐SceI site on the X chromosome were infected with lentiviral vectors expressing I‐SceI‐GR or empty vector. Then, the cells were treated with doxycycline (Dox) for inducing I‐SceI‐GR expression. Next, acetonide (TA) was added to the cells to induce the translocation of I‐SceI from cytoplasm into the nucleus for generating a solo DSB in each cell. The samples were then subjected to ChIP assay. ChIP assays were described previously.^[^ ^91^, ^92^ ^]^ Briefly, samples were immunoprecipitated with TopBP1, γH2AX, rRNA, or FBL antibodies following the ChIP protocol. Finally, DNA was extracted with phenol‐chloroform. After isopropanol‐precipitated in the presence of glycogen (Thermo Fisher Scientific), DNA was dissolved in nuclease‐free water. qPCR was performed in triplicate. Sequences of the primers were:

500BP‐S, CTCTTCAGCAATATCACGGGTAGC;

500BP‐AS, TGGACGAAGAGCATCAGG.

### Stellaris RNA FISH and Immunofluorescence Staining for Somatic Cells

Stellaris RNA FISH and immunofluorescence were performed according to the manufacturer's protocol.^[^ ^55^ ^]^ Briefly, cells were grown on glass coverslips and treated with IR. After washing with PBS, the cells were treated with HEB buffer for 30 min, then, in addition to adding sucrose solution for 30 min. The cells were fixed with 2% paraformaldehyde and permeabilized with 0.2% Triton X‐100 in PBS for 30 min at room temperature. After washing with PBS, cells were rehydrated with wash buffer A at room temperature for 5 min. Then the cells were incubated with hybridization buffer with 12.5 pmol RNA FISH pre‐rRNA probe sets in a humidified chamber overnight at 37 °C. Then cells were washed once by wash buffer A at room temperature for 10–30 min and subjected to immunofluorescence staining. After washing with PBS, the cells were then incubated with primary antibody, which were diluted in 3% BSA. The cells were then washed with PBST (PBS with 0.2% Tween 20) and incubated with secondary antibody. Then, the cells were then washed with PBST (PBS with 0.2% Tween 20). The coverslips were mounted, and the cells were imaged on an LSM 800 confocal Airyscan (Zeiss, Oberkochen, Germany). For RNA polymerase inhibitor treatment, Pol I inhibitor BMH‐21 (1 µm) was added to the cell culture medium 2 h before IR. Then, 24 h after IR, the cells were fixed and stained with indicated antibodies for immunofluorescence staining. Processing and quantitative measurements of fluorescence intensities were performed with ImageJ. DNA damage foci were counted by FociPicker3D.

RNA FISH pre‐rRNA probes were:

Human‐ITS1‐Cy3, CCTCGCCCTCCGGGCTCCGTTAATGATC;

Human‐ITS2‐Cy3, CTGCGAGGGAACCCCCAGCCGCGCA.

### Harvesting Spermatogonia, Meiotic Spreads of Spermatocytes

These experimental procedures have been described previously.^[^ ^55^ ^]^ Briefly, spermatogonia were isolated from 3‐week‐old mice. Then, the cells were cultured in Dulbecco's modified Eagle medium (DMEM) supplemented with 10% FBS, 1% penicillin, and 1% streptomycin at 37 °C with 5% CO_2_. For meiotic spreads of spermatocytes, the cells were resuspended with freshly prepared Methanol/Acetic Acid fixation fixative (Methanol:Acetic Acid = 3:1) or paraformaldehyde fixation fixative. Finally, the cells were dropped onto glass slides and air‐dried.

### RNA FISH and Immunofluorescence Staining for Meiotic Spreads

Similar to procedures used in somatic cells, Stellaris RNA FISH and immunofluorescence staining were performed according to the manufacturer's protocol. Briefly, slides were rehydrated with wash buffer A (SMF‐WA1‐60, Bioresearch technologies, USA) at room temperature for 5 min. Then, the slides were incubated with hybridization buffer (SMF‐HB1‐10, Bioresearch technologies, USA) with 12.5 pmol RNA FISH probe overnight at 37 °C. The slides were washed once by wash buffer A at room temperature for 10–30 min and subjected to immunofluorescence staining. Of note, the immunofluorescence staining was performed after RNA FISH staining to detection of both RNAs and proteins. RNA FISH pre‐rRNA probes were:

Mouse‐ITS1‐Cy3, ACGGGTCAAAAACCCGTAACGACGTA,

CCCCAGCCAACGTAGAAAAGCCAGA,

AAAAAAGGGTCGGGAGCGGAAAAACA,

AGCAGGAACGAAACGAGACACGTGT;

Mouse‐ITS2‐Cy3, GGTTGGCCCTGCGAGCAAACTCCCAGCCG,

ACCGCCCACACGTCTGAACTTCGGGAGAC,

CCGCAGGGGACCGACCACCACCTCGGCG.

### RNase A Treatment, RNase H Treatment, GST‐Protein Hybridization, and Pre‐rRNA Blocking Assays

For RNase A treatment or RNase H treatment, the cells were permeabilized with 0.2% Triton X‐100 in PBS for 5 min at room temperature. Then 200 µg mL^−1^ RNase A (NEB) or 100 U mL^−1^ RNase H (NEB) was applied to the cells for 1 h at room temperature and washed three times with PBS. Then, the cells were fixed with 2% paraformaldehyde to IF or GST‐protein hybridization. For GST‐protein hybridization, 0.2 µg µL^−1^ recombinant GST‐proteins were added to the cells and incubated for 1 h at room temperature. After washing with PBS, the cells were then stained with the GST and γH2AX antibodies. For pre‐rRNA blocking, 1 µg µL^−1^ of pre‐rRNA was pre‐incubated with 0.2 µg µL^‐1^ GST‐proteins for 30 min at room temperature and then subjected to the GST‐protein hybridization process.

### RNA Polymerase Inhibitors Treatment and ATM Kinase Inhibitor Treatment Assays

For RNA polymerase inhibitors treatment, Pol I inhibitor (BMH‐21) (1 µm), Pol II inhibitor (α‐amanitin) (10 µg mL^−1^), or Pol III inhibitor (ML‐60218) (10 µm) was added to the cell culture medium for 2 h before ionizing radiation. For ATM kinase inhibitor treatment, KU‐55933 (Selleck) (10 µm) was added to the cell culture medium 2 h before ionizing radiation. KU‐60019 (Selleck) (3 µm) was added to the cell culture medium 6 h before ionizing radiation. For all treatments, 2 h after IR, the cell culture medium was changed.

### In Vitro GST Pulldown Assay and BLI Assays

Terminal GST‐tagged TopBP1‐BRCT4‐5 were expressed in BL21(DE3)pLysS (Promega) followed by protein purification with Glutathione Sepharose 4B (17‐0756‐05, GE Healthcare). Purified GST‐TopBP1‐BRCT4‐5 proteins were incubated with total RNA at 4 °C for 2 h. Then, Glutathione Sepharose 4B beads were added to the reaction mixture and incubated for 1.5 h at 4 °C. The beads were washed three times with NETN100 at 4 °C, and the supernatant was discarded. PK buffer was added to the beads and incubated for 10 min at 37 °C. The same volume of Urea/PK buffer was subsequently added to the mixture and sequentially incubated for 10 min at 37 °C. After incubating with the buffer, the mixture was centrifuged at 4 °C and the supernatant was discarded. RNA was precipitated with ethanol in the presence of glycogen (Thermo Fisher Scientific). Then, cDNA was prepared from precipitated RNA using SuperScript III and random primers (Invitrogen). RT‐qPCR was performed in triplicate.

For BLI assay, purified GST‐TopBP1‐BRCT4‐5 and Biotin‐pre‐rRNA fragments were added to the PBST buffer (PBS, 0.2% Tween 20) with indicated concentrations. Biotin‐pre‐rRNA fragment was chemically synthesized (Tsingke):

25 nt, AUCGACACUUCGAACGCACUUGCGG;

6 nt, CGCACU;

3 nt, GGG.

### Northern Blotting

Precipitated RNA from in vitro pulldown assay was subjected to northern blotting. Briefly, RNA was first separated on 1.5% denatured agarose gel in MOPS buffer, then transferred to positively charged Amersham Hybond‐N+ membranes (GE Healthcare) in 20× SSC buffer. Then, the membranes were cross‐linked with 0.12 J cm^−2^ 254 nm UV and dried at 80 °C for 30 min. After pre‐hybridization with Ambion ULTRAhyb‐Oligo buffer for 30 min, the membranes were hybridized with different Biotin‐labeled probes for at least 3 h. Finally, the signal was detected with the Chemiluminescent Nucleic Acid Detection Module Kit (Thermo Fisher Scientific). The Biotin‐labeled probes were:

ITS1‐Biotin, CCTCGCCCTCCGGGCTCCGTTAATGATC;

ITS2‐Biotin, CTGCGAGGGAACCCCCAGCCGCGCA.

### Co‐Immunoprecipitation (Co‐IP) and Western Blotting

HEK293T cells stably expressing SFB‐TopBP1 were treated with or without IR. Twenty‐four hours after IR, the cells were lysed in 0.5% Triton X‐100 lysis buffer (20 mm Tris‐Cl, pH 7.5, 150 mm NaCl, 0.5% Triton X‐100, 10% glycerol, and 1 mm EDTA) in the presence of DTT, protease inhibitor cocktail (Roche), phosphatase inhibitor cocktail (Roche), RNase inhibitor, VRC and 25 U mL^−1^ DNase I (Beyotime). Then, the cell lysates were incubated with anti‐Flag beads (Sigma) for 6 h at 4 °C. The beads were washed three times with lysis buffer containing 300 mm NaCl. For RNase A treatment, RNase A (NEB) (200 µg mL^−1^) was applied to the beads in lysis buffer for 1 h at 4 °C. Then, the beads were washed three times with lysis buffer. The samples were analyzed by Western blotting with the indicated antibodies.

### In Vitro Phase Separation Assay and FRAP

In vitro phase separation assay was performed in the phase separation buffer (20 mm Tris‐HCl, pH 7.5, 100 mm NaCl, 5 mm KH_2_PO_4_, 1.5 mm MgCl_2_, and 1 mg mL^−1^ BSA). Purified GFP‐TopBP1‐BRCT4‐5 and pre‐rRNA were added to the buffer with indicated concentrations, and PEG8000 (NEB) (final concentration, 5% (w/v)) was also added. The mixture was directly pipetted onto glass‐bottomed dishes (NEST), and observed by a Nikon confocal microscope with 40× differential interference contrast (DIC). The method used for pre‐rRNAs isolated from 293T cells has been described previously.^[^ ^55^ ^]^

For in vitro FRAP experiments, liquid droplets photobleached with 80% laser power for 1 s using 488‐nm lasers were performed on a Nikon confocal microscope with 40× differential interference contrast (DIC).

### GFP Reporter Assays

This system was described previously.^[^ ^91^, ^93^ ^]^ Briefly, DR‐GFP‐U2OS cells were transfected with the I‐SceI‐GR plasmid. Then, the cells were treated with doxycycline and TA to induce DSBs. Two days after treatment, the efficiency of homologous recombination repair was examined by FACS.

### Cell Cycle Checkpoint Assays

For the transient G2/M transition checkpoint assay, 2 h before IR, the cells were treated with RNA polymerase inhibitors. One hour after 2 Gy of IR, the cells were fixed with 70% ethanol and permeabilized with 0.2% Triton X‐100 in PBS for 10 min at room temperature. After washing with PBS, the cells were then incubated with anti‐Histone H3 pSer10 antibody, which was diluted in 3% BSA. After washing, the cells were incubated with FITC secondary antibody. Finally, the cells were treated with RNase A for 10 min at room temperature, and incubated with propidium iodide. The pSer10 (pH3) positive cells were examined by FACS.

For 5‐ethynyl‐2′‐deoxyuridine (EdU)‐labeling experiment, 2 h before IR, the cells were treated with RNA polymerase inhibitors. After 10 Gy of IR, the cells were incubated with 10 µm EdU for 30 min. Then, the cells were fixed with cold 70% ethanol at 4 °C overnight, which can be stored for up to 1 week. Then, the Click‐iT EdU Alexa Fluor 488 Flow Cytometry Assay Kit (Invitrogen, C10425) and FxCycle Violet Stain (Invitrogen, F10347) were used. FACS was performed to examine DNA synthesis of S phase through median EdU intensity.

### Statistical Analysis

All results were analyzed using GraphPad Prism 5.0 software and Microsoft Excel. The representative images of IF staining and Western blotting are shown. The data are shown as means ± S.D. Significant differences between the two groups were performed using two‐tailed Student's *t*‐test. For all experiments, *p*‐values < 0.05 was considered statistically significant.

## Conflict of Interest

The authors declare no conflict of interest.

## Author Contributions

D.X., X.G., and Y.M. contributed equally to this work. X.Y. designed the project. D.X., X.G., Y.M., Z.L., and Q.L. performed the experiments. All the authors analyzed the data. D.X. and X.Y. wrote the manuscript. All authors reviewed the results and approved the final version of the manuscript.

## Supporting information

Supporting Information

## Data Availability

The data that support the findings of this study are available from the corresponding author upon reasonable request. All datasets have been deposited in the GEO Datasets under the GEO accession number GSE213073 and GSE182987.
