# Supplementary material for: Pre‐rRNA Facilitates TopBP1‐Mediated DNA Double‐Strand Break Response
Source: Adv Sci (Weinh). 2023 Aug 15;10(28):2206931. doi: 10.1002/advs.202206931 (PMC10558638; doi:10.1002/advs.202206931)
Supplement: Supplementary file 1 — Supporting Information [file ADVS-10-2206931-s001.pdf]

ADVANCED SCIENCE
Open Access

Supporting Information

for *Adv. Sci.*, DOI 10.1002/advs.202206931

Pre-rRNA Facilitates TopBP1-Mediated DNA Double-Strand Break Response

*Di Xin, Xiaochen Gai, Yidi Ma, Zexing Li, Qilin Li and Xiaochun Yu**

Supplemental Figure S1

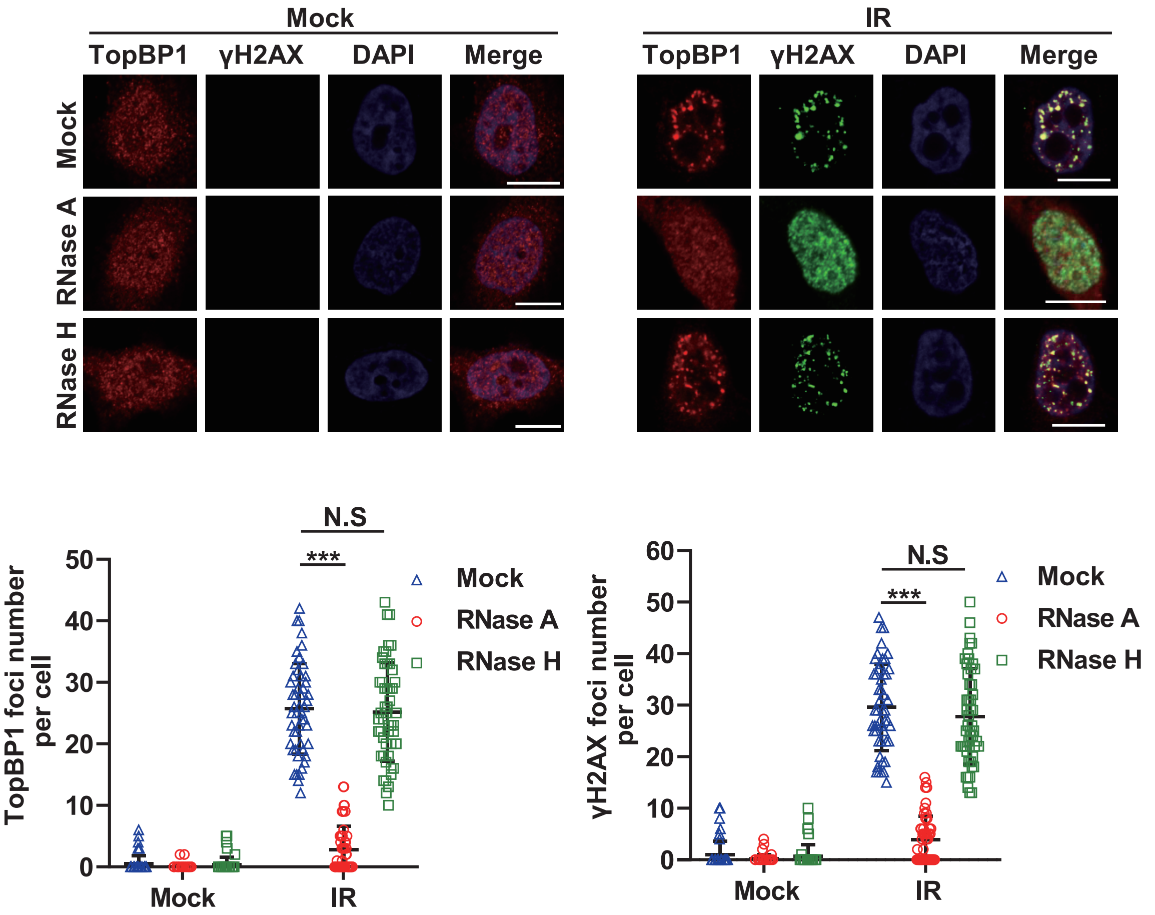

Supplemental Figure S1. The RNase A treatment impairs IRIF of TopBP1. Following 10 Gy of IR, HCT116 cells were treated with RNase A or RNase H. IRIF of TopBP1 and γH2AX was examined by anti-TopBP1 and anti-γH2AX antibodies (upper panels). The foci in each cell were counted (lower panels). The bars represent the mean values ± SD (n = 50 from three independent experiments, per group). Two-tailed student's t test is used to determine statistical significance. ***, p < 0.001; N.S, not significant, versus control groups. Scale bars, 10 μm.

Supplemental Figure S2

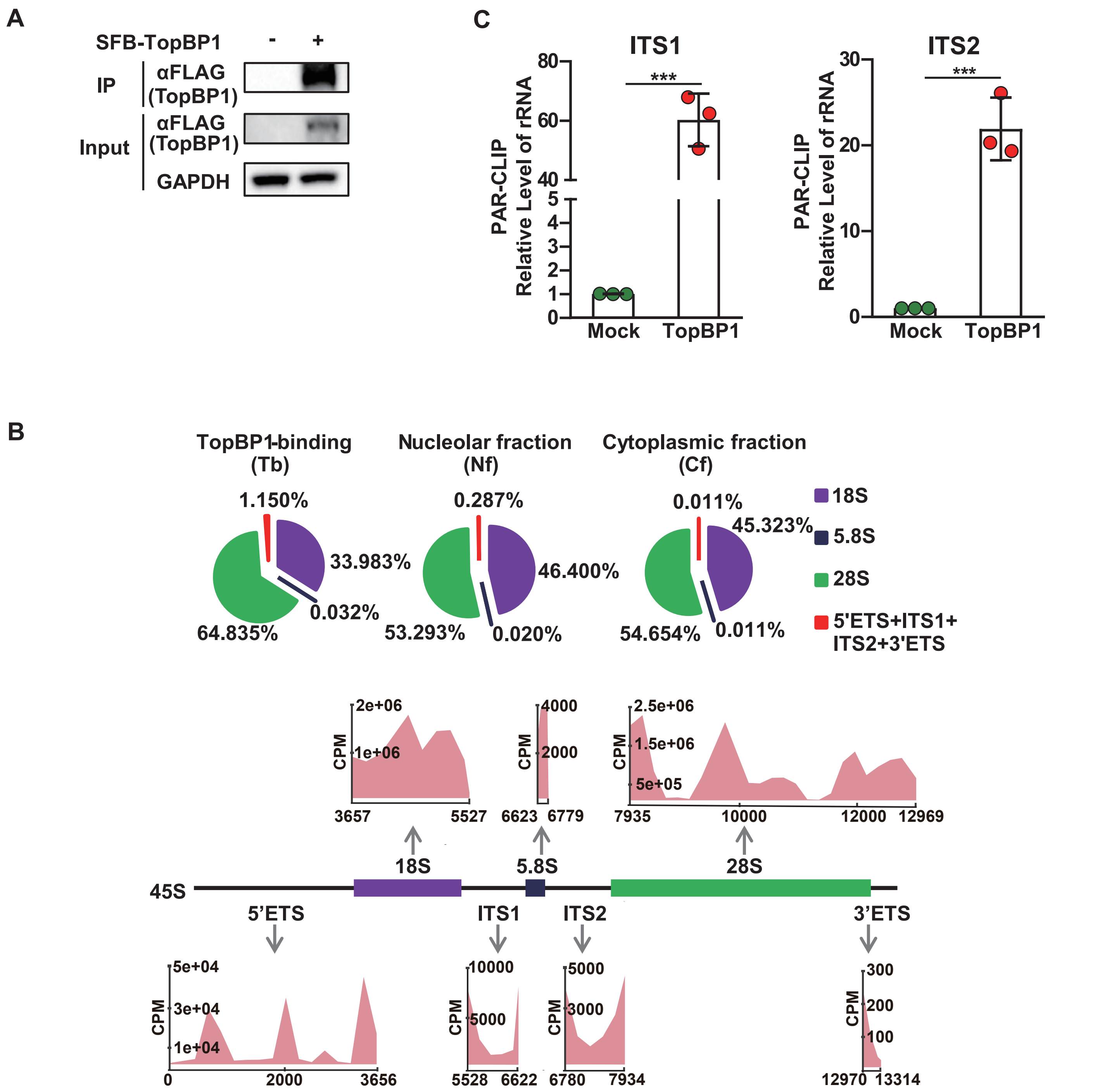

Supplemental Figure S2. TopBP1 is associated with pre-rRNA. (**A**) 293T cells stably expressing SFB-TopBP1. Immunoblotting with the indicated antibodies was performed to examine the expression of SFB-TopBP1. (**B**) TopBP1-associated RNA covers the whole 45S pre-rRNA region. The RNA sequencing reads amount from TopBP1-binding, nucleolar fraction or cytoplasmic fraction in each region of 45S pre-rRNA were compared (upper panels). Distribution of TopBP1-bound rRNA on 45S rDNA locus (lower panels). (**C**) TopBP1 is associated with pre-rRNA. The association of pre-rRNA with TopBP1 was validated using RT-qPCR following PAR-CLIP. Data are represented as means ± SD as indicated from three independent experiments. Two-tailed student's *t* test is used to determine statistical significance. ***, $p < 0.001$, versus control groups.

Supplemental Figure S3

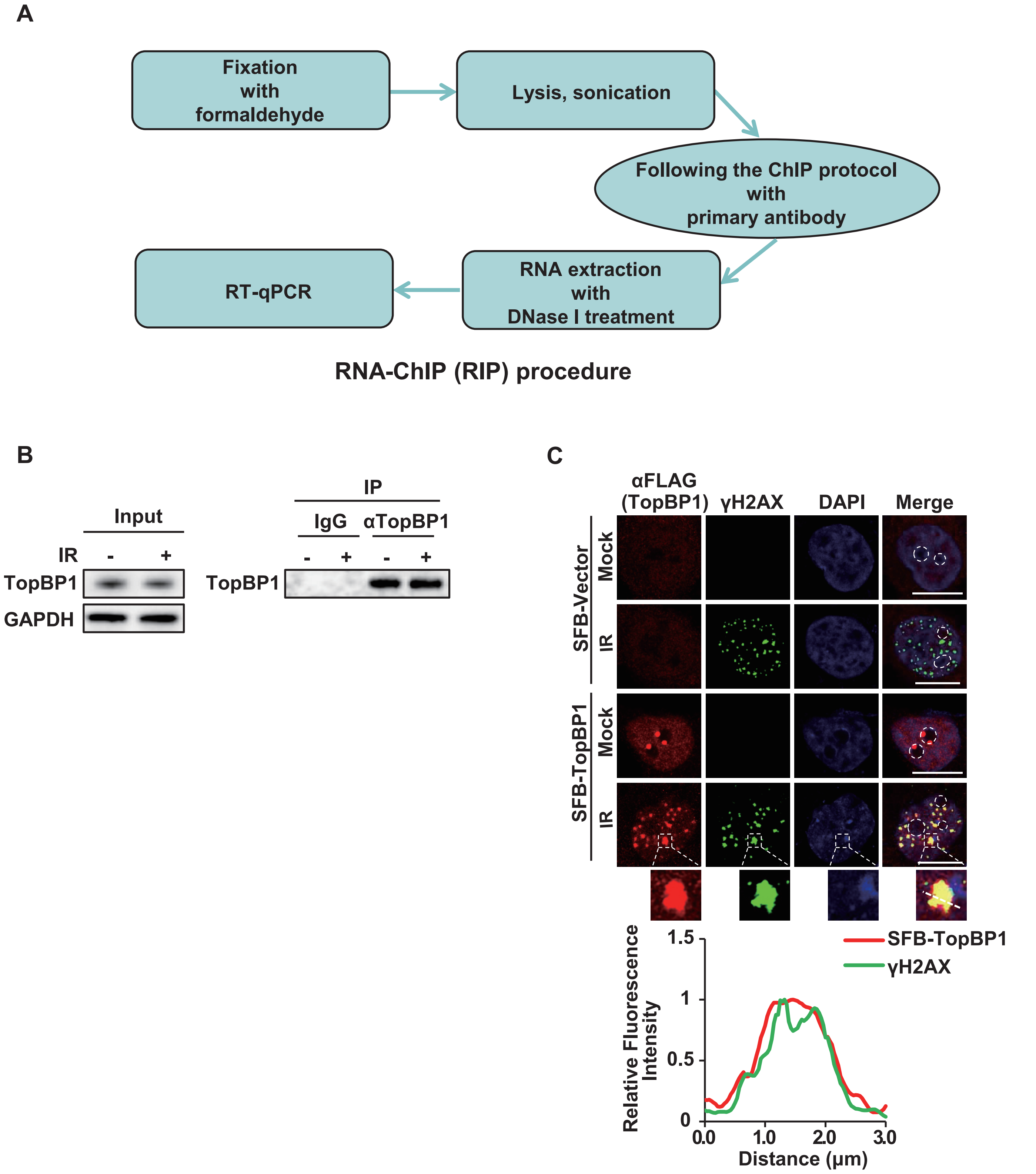

Supplemental Figure S3. RNA-ChIP assays to identify TopBP1-associated RNA. (**A**) A diagrammatic representation showing the procedure of RNA-ChIP (RIP). (**B**) The expression of endogenous TopBP1 is not altered by IR treatment. The cell lysates were immunoprecipitated with anti-TopBP1 antibody, followed by immunoblotting with anti-TopBP1 antibody (right panel). Protein inputs were shown in the left panel. (**C**) SFB-TopBP1 forms nuclear foci and localizes to the DSB sites following IR.HEK293T cells stably expressing SFB-TopBP1 or SFB-vector were treated with or without 10 Gy of IR. Then, IF was performed with anti-FLAG and anti-γH2AX antibodies (upper panels). The relative intensity of fluorescence signals is analyzed (lower panel). Nucleoli are marked by circular dashed lines. Scale bars, 10 μm.

Supplemental Figure S4

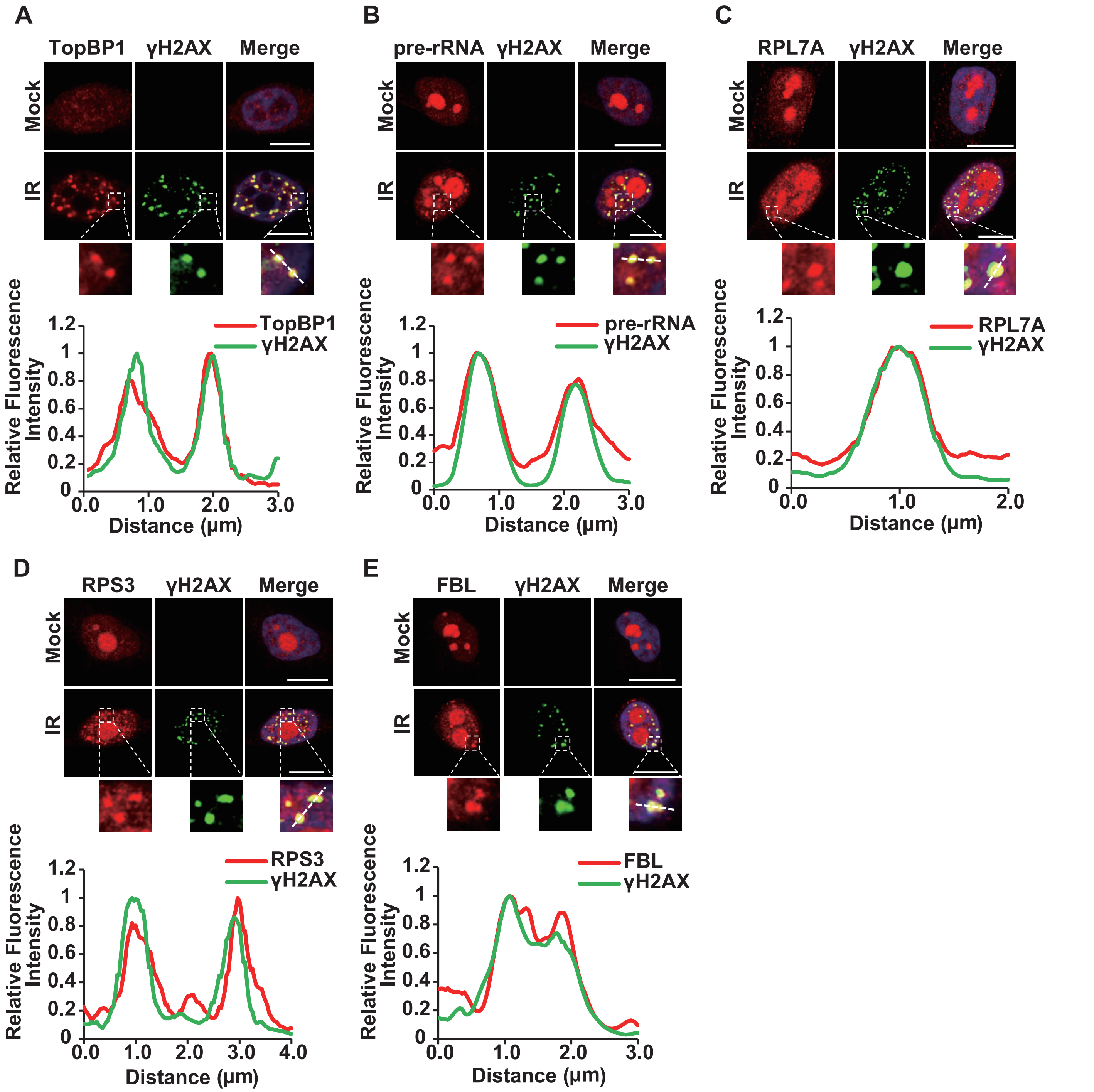

Supplemental Figure S4. Pre-rRNPs colocalize with γH2AX at DSBs. (**A**) γH2AX colocalizes with TopBP1 at DSBs. Following 10 Gy of IR treatment on HCT116 cells, IF was performed with anti-TopBP1 and anti-γH2AX antibodies. (**B**) Pre-rRNA colocalizes with γH2AX at DSBs. Following 10 Gy of IR treatment on HCT116 cells, RNA FISH with pre-rRNA probes and IF with anti-γH2AX antibody were performed. (**C-E**) Pre-rRNA-associated proteins colocalize with γH2AX at DSBs. Following 10 Gy of IR, IF was performed with anti-γH2AX and anti-RPL7A (**C**), or anti-RPS3 (**D**), or anti-FBL antibodies (E). The relative intensity of fluorescence signals is analyzed. Scale bars, 10 μm.

Supplemental Figure S5

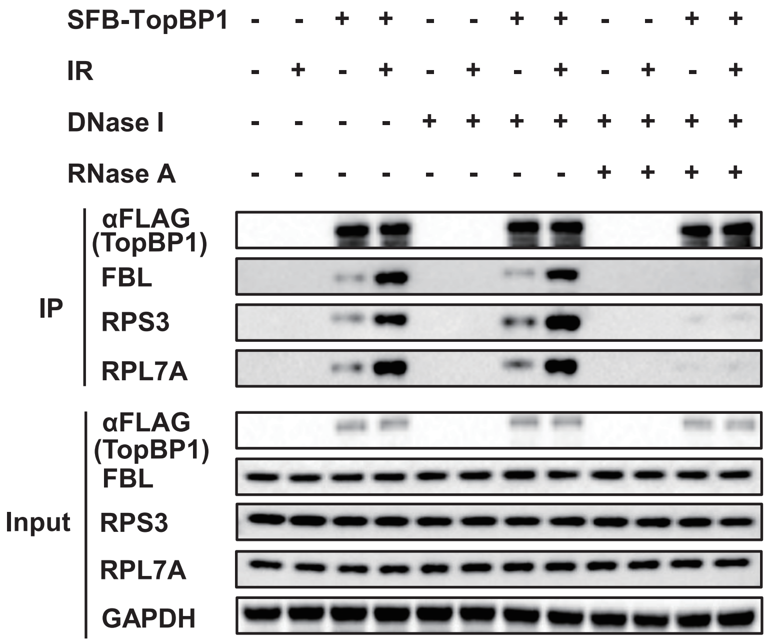

Supplemental Figure S5. TopBP1 interacts with pre-rRNA-associated proteins. HEK293T cells stably expressing SFB-TopBP1 were treated with or without 10 Gy of IR. Following with or without DNase I treatment, cell lysates were immunoprecipitated with anti-FLAG beads. The beads were treated with or without RNase A. Immunoblotting was performed with the indicated antibodies.

Supplemental Figure S6

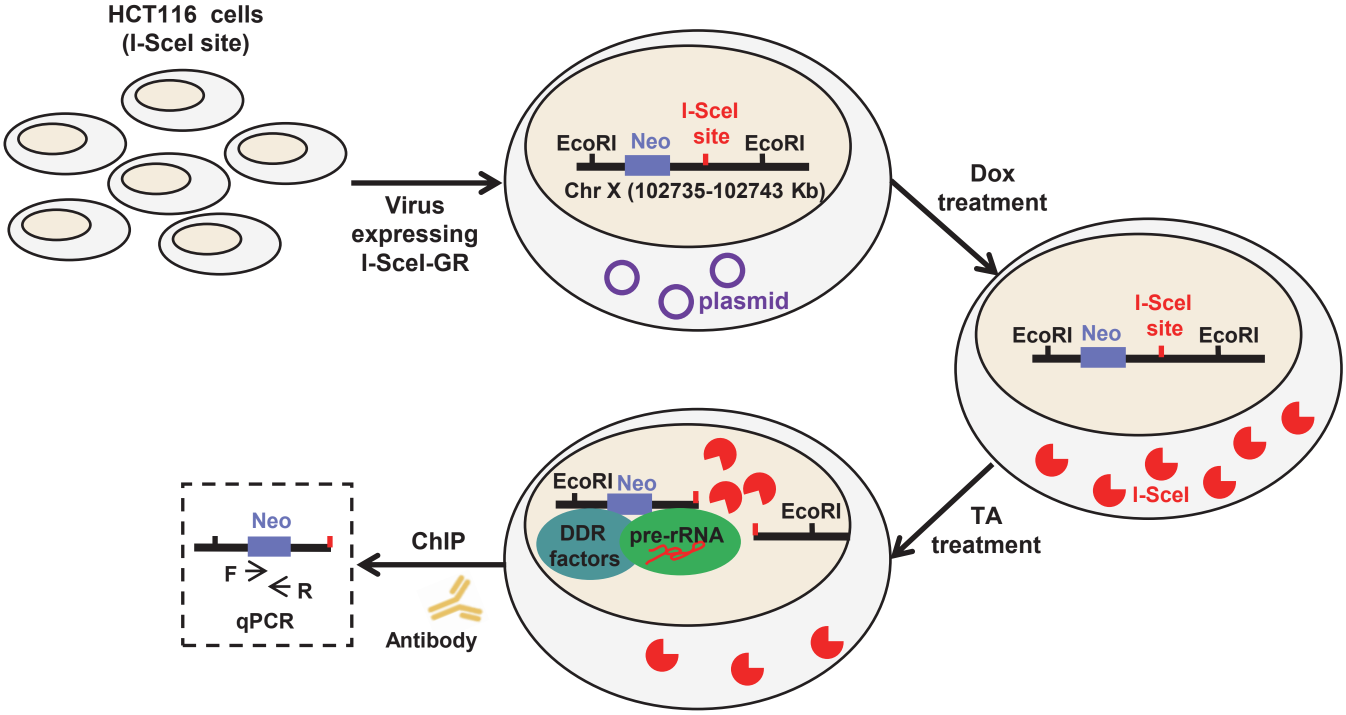

Supplemental Figure S6. Diagrammatic representation showing the procedure of I-SceI-induced solo DSB system. I-SceI is induced to express and translocate from cytoplasm to nucleus, where it creates a solo DSB. ChIP is performed with primary antibodies.

Supplemental Figure S7

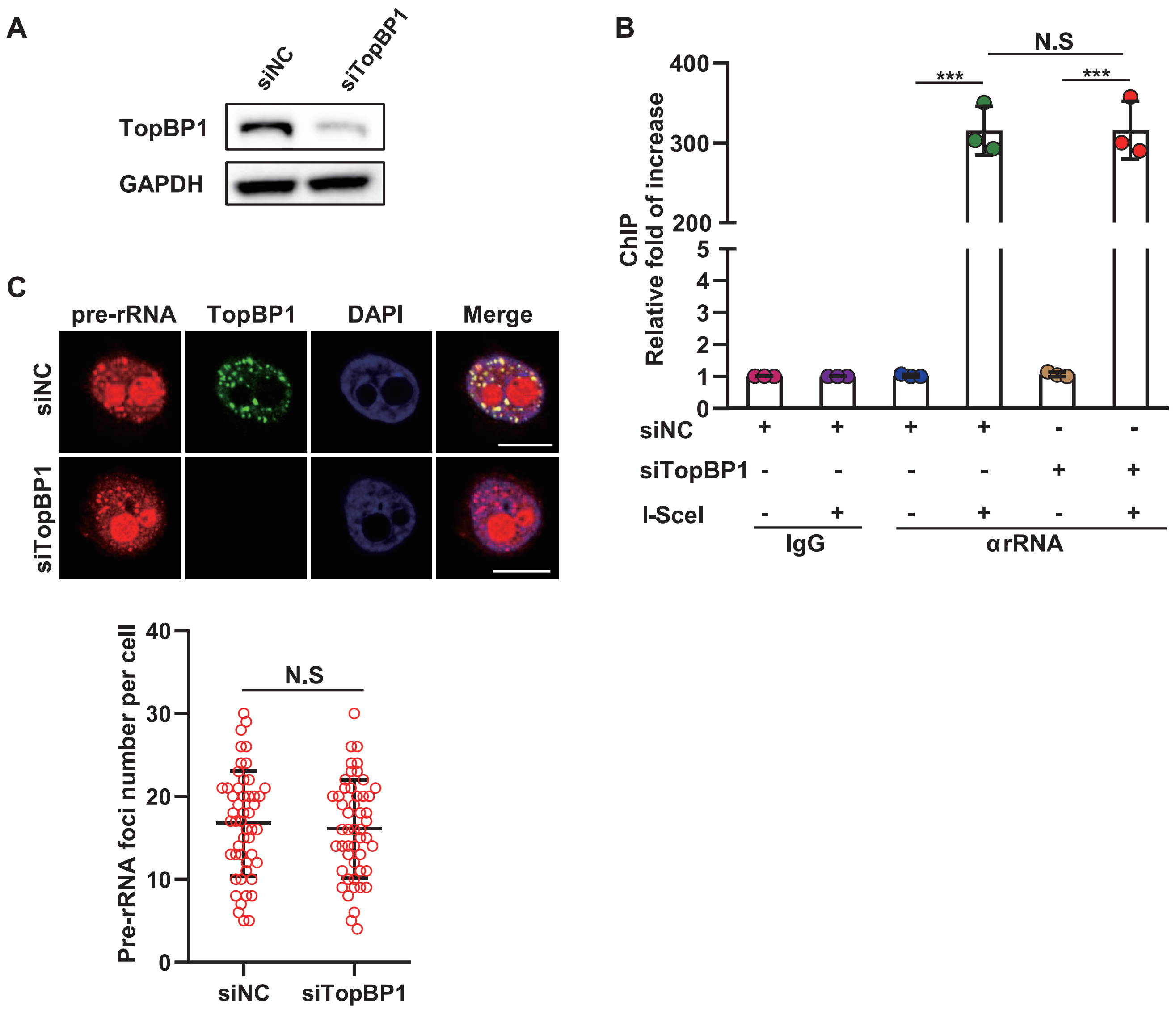

Supplemental Figure S7. TopBP1 does not affect the loading of pre-rRNA to DSBs. (**A**) siRNA treatment specifically down-regulates the expression of TopBP1. (**B**) Depletion of TopBP1 does not affect the accumulation of pre-rRNA to DSBs. The accumulation of rRNA at the DSB was examined by ChIP using the I-SceI-induced solo DSB system. Data are represented as means ± SD as indicated from three independent experiments. (**C**) Depletion of TopBP1 does not affect the IRIF of pre-rRNA. IRIF of pre-rRNA was examined by RNA FISH with pre-rRNA probes (upper panels). The foci in each cell were counted (lower panel). The bars represent the mean values ± SD ($n = 50$ from three independent experiments, per group). Two-tailed student's t test is used to determine statistical significance. ***, $p < 0.001$; N.S, not significant, versus control groups. Scale bars, 10 μm.

Supplemental Figure S8

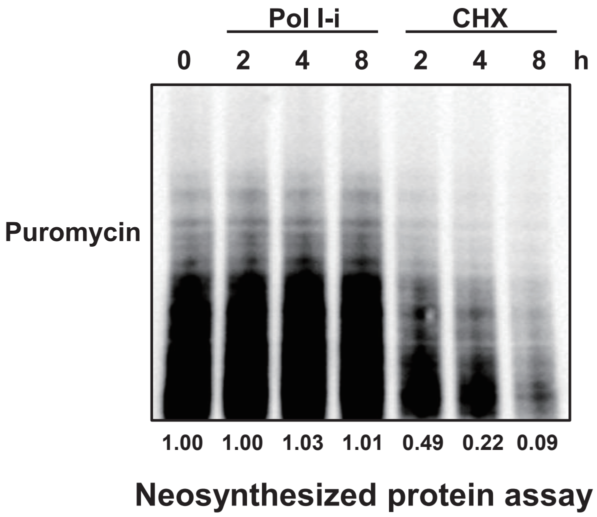

Supplemental Figure S8. Transient RNA Pol I inhibitor treatment does not affect protein translation. After BHM-21 (Pol I inhibitor) or Cycloheximide (CHX, translational elongation inhibitor) treatment, HCT116 cells were incubated with puromycin for 30 minutes. The neosynthesized protein was examined by immunoblotting using anti-puromycin antibody. Numbers below lanes indicate the relative densitometry of the protein presented.

Supplemental Figure S9

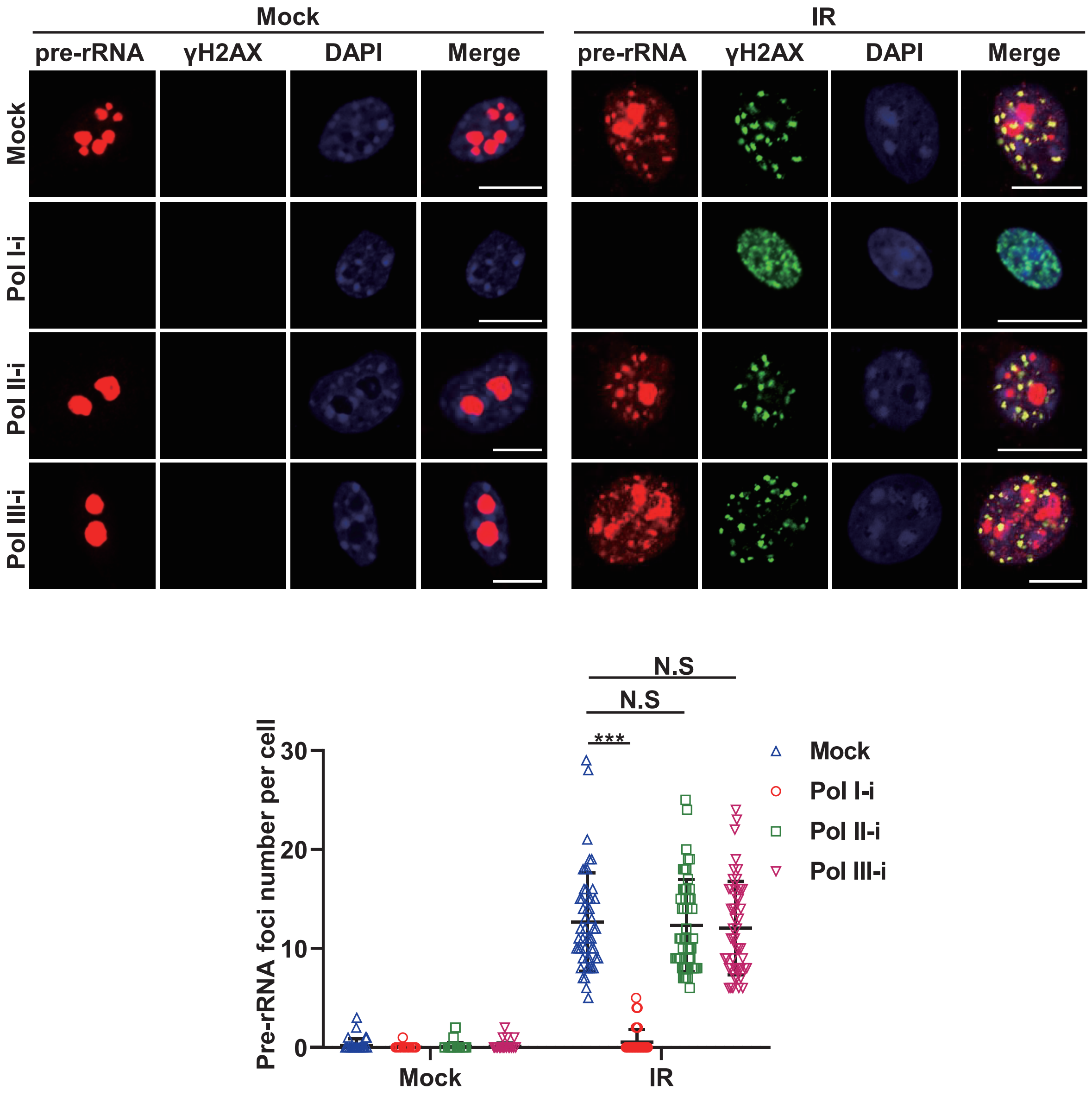

Supplemental Figure S9. RNA Pol I inhibitor treatment affects the pre-rRNA foci at DSBs. Following Pol I, Pol II or Pol III inhibitor treatment, MEFs were treated with or without 10 Gy of IR. RNA FISH with pre-rRNA probes and IF with anti-γH2AX antibody were performed (upper panels). Foci number in each cell was counted (lower panel). The bars represent the mean values ± SD ($n = 50$ from three independent experiments, per group). Two-tailed student's t test is used to determine statistical significance. ***, $p < 0.001$; N.S, not significant, versus control groups. Scale bars, 10 μm.

Supplemental Figure S10

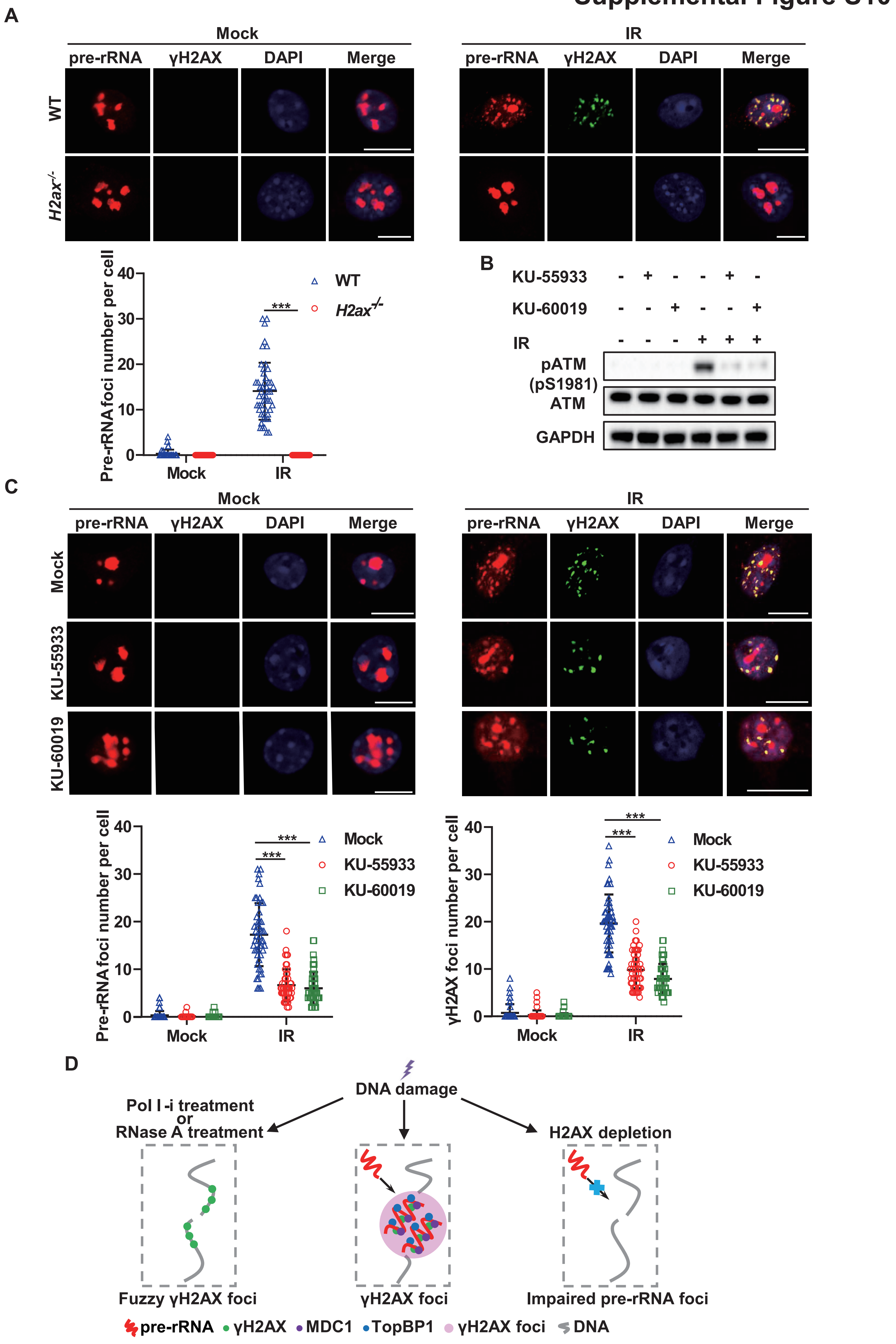

Supplemental Figure S10. The foci formation of pre-rRNA is dependent on ATM-γH2AX axis. (**A**) Detection of pre-rRNA and γH2AX foci in WT and *H2ax*$^{-/-}$ MEFs. Following with or without 10 Gy of IR treatment on MEFs, RNA FISH with pre-rRNA probes and IF with anti-γH2AX antibody were performed (upper panels). Foci number in each cell was counted (lower panel). The bars represent the mean values ± SD ($n = 50$ from three independent experiments, per group). (**B**) and (**C**) ATM inhibitors treatment reduces the foci formation of γH2AX and pre-rRNA. Following with or without ATM inhibitors treatment, MEFs were treated with or without 10 Gy of IR. Immunoblotting with the indicated antibodies was performed to examine the level of pATM (**B**). RNA FISH with pre-rRNA probes and IF with anti-γH2AX antibody were performed (**C**) (upper panels). Foci number in each cell was counted (lower panels). The bars represent the mean values ± SD ($n = 50$ from three independent experiments, per group). (**D**) A working model of the functional interaction between γH2AX-MDC1 and pre-rRNA mediates DSB-induced foci formation. A positive feedback loop may form between γH2AX and pre-rRNA. H2AX is phosphorylated by ATM for the recruitment of its functional partner MDC1. Once MDC1 is recruited, it associates with pre-rRNA, which acts as a scaffold to maintain DSB-induced foci (including γH2AX foci) and recruit downstream factors such as TopBP1 to DSBs. Two-tailed student's *t* test is used to determine statistical significance. ***, $p < 0.001$, versus control groups. Scale bars, 10 μm.

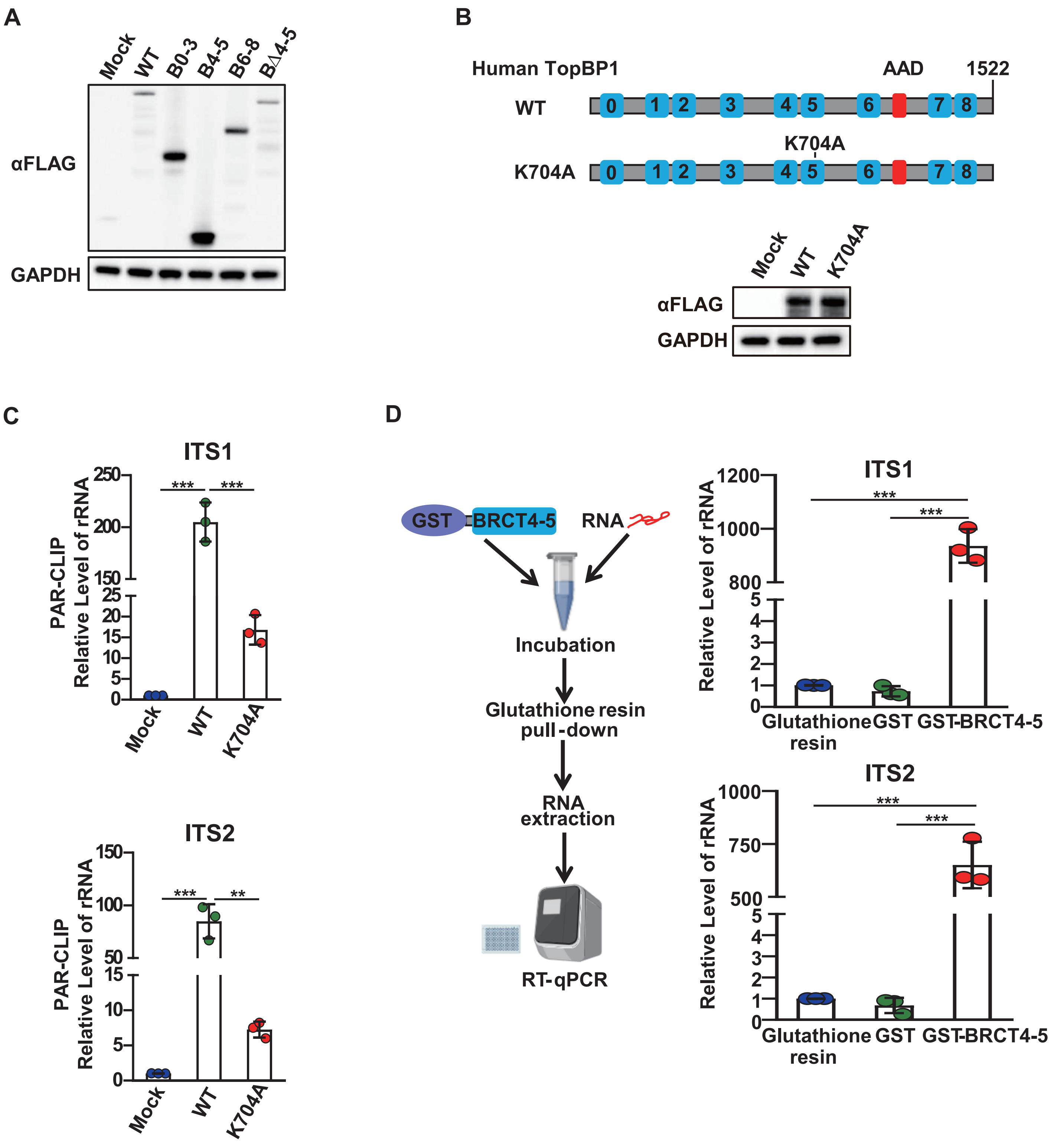

Supplemental Figure S11. Characterization of the TopBP1–pre-rRNA interaction. (**A**) Immunoblotting with the indicated antibodies was performed to examine the SFB-tagged full-length and deletion mutants of TopBP1. (**B**) Generation of the K704A mutant. The expression of the K704A mutant was examined by Western blotting. (**C**) The K704A mutation largely impairs the interaction with pre-rRNA. PAR-CLIP assays were performed. The relative levels of pre-rRNA were shown. (**D**) The BRCT4-5 domain of TopBP1 interacts with pre-rRNA. The procedure of pull down and RT-qPCR assay is shown in the left panels. The enrichment of pre-rRNA is shown in the right panels. Data are represented as means ± SD as indicated from three independent experiments. Two-tailed student's *t* test is used to determine statistical significance. **, $p < 0.01$; ***, $p < 0.001$, versus control groups.

Supplemental Figure S12

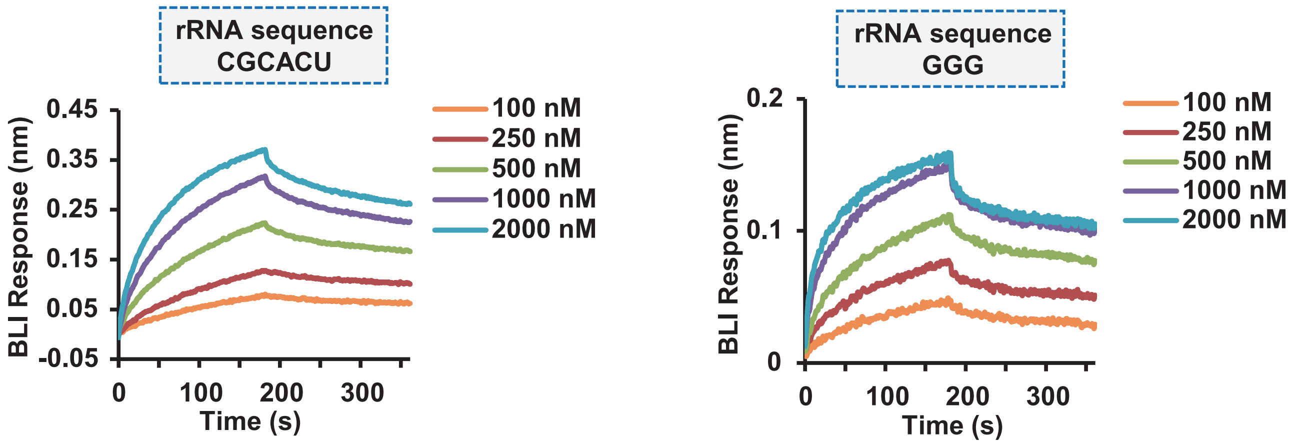

Supplemental Figure S12. Recombinant TopBP1 BRCT4-5 binds to RNA oligos. The binding affinity between the recombinant TopBP1 BRCT4-5 and biotin-labeled pre-rRNA oligos was analyzed by BLI.

Supplemental Figure S13

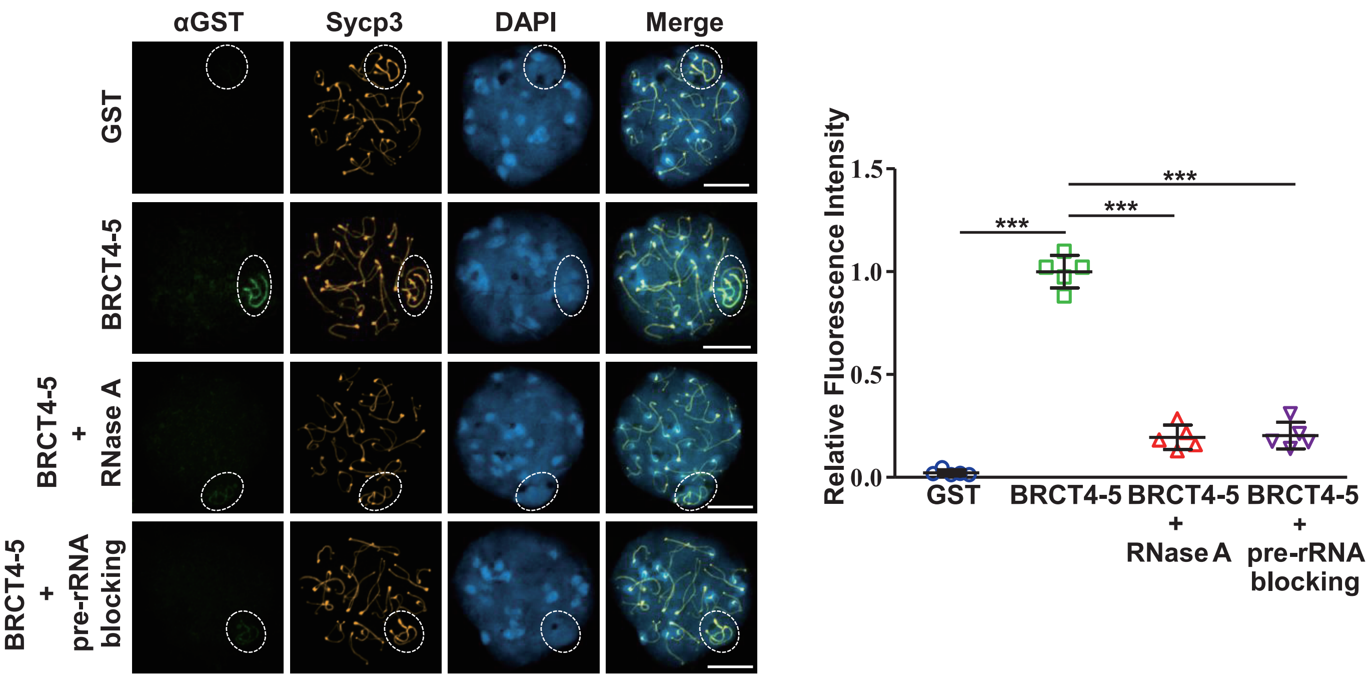

Supplemental Figure S13. TopBP1 BRCT4-5 recognizes pre-rRNA in the XY body. Following RNase A treatment or pre-rRNA blocking, protein hybridization assay was performed (left panels). The relative signal intensity of TopBP1 BRCT4-5 in the XY body is statistically analyzed (right panel). Two-tailed student's *t* test is used to determine statistical significance. ***, $p < 0.001$, versus control groups. Scale bars, 10 μm.

A

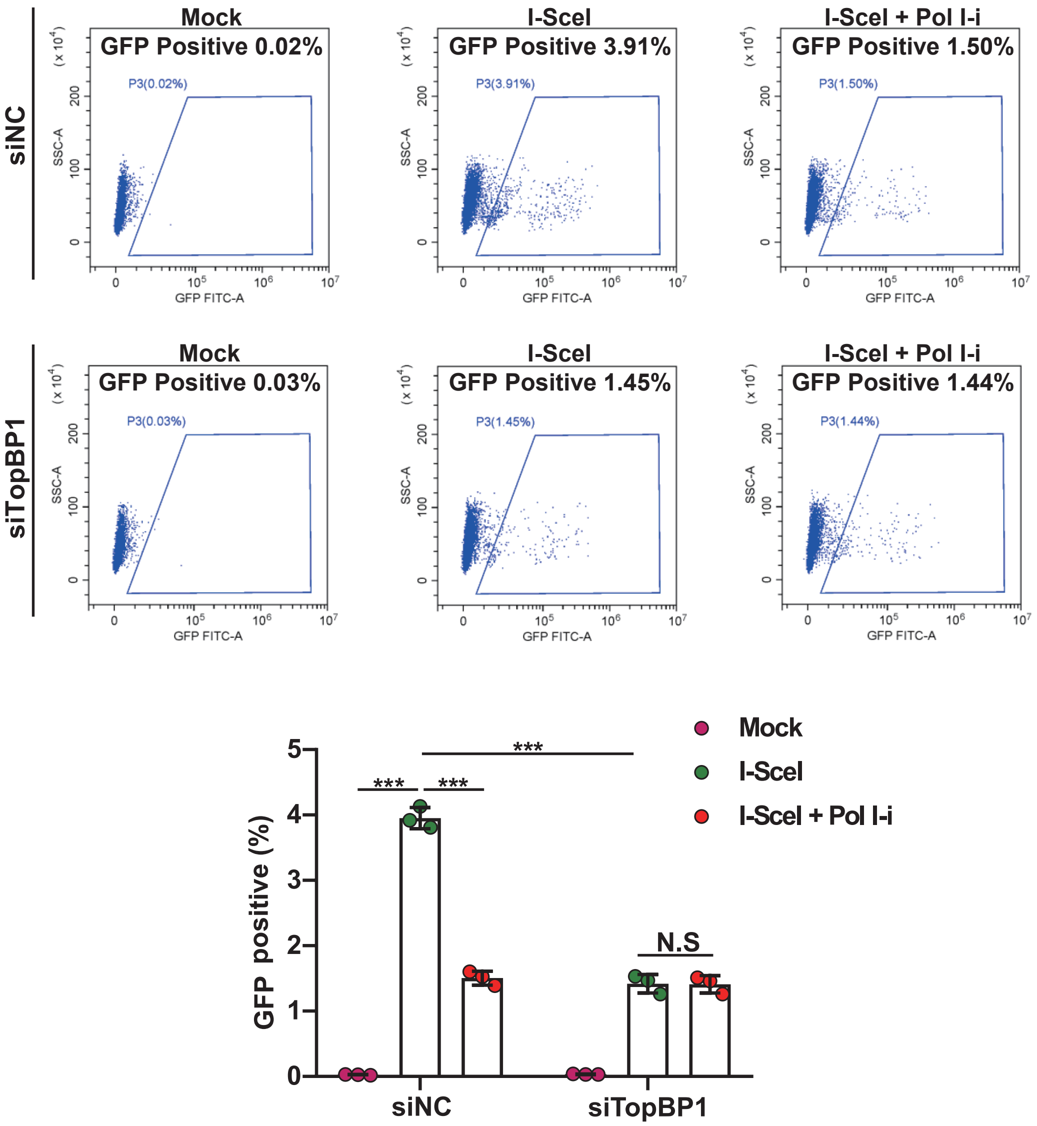

B

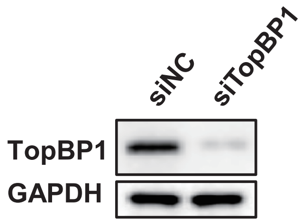

Supplemental Figure S14. Pre-rRNA regulates TopBP1-mediated HR repair. (**A**) Pol I inhibitor treatment suppresses HR repair. First, DR-GFP-U2OS cells were transfected with siNC or siTopBP1 oligos. Then, following Pol I, Pol II or Pol III inhibitor treatment, DR-GFP-U2OS cells were used to measure the HR efficiency, and GFP positive population was examined by flow cytometry (upper panels). The percentage of GFP positive cells was statistically analyzed (lower panel). Data are represented as means ± SD as indicated from three independent experiments. (**B**) siRNA treatment specifically down-regulates the expression of TopBP1. Two-tailed student's *t* test is used to determine statistical significance.***, $p < 0.001$; N.S, not significant, versus control groups.

Supplemental Figure S15

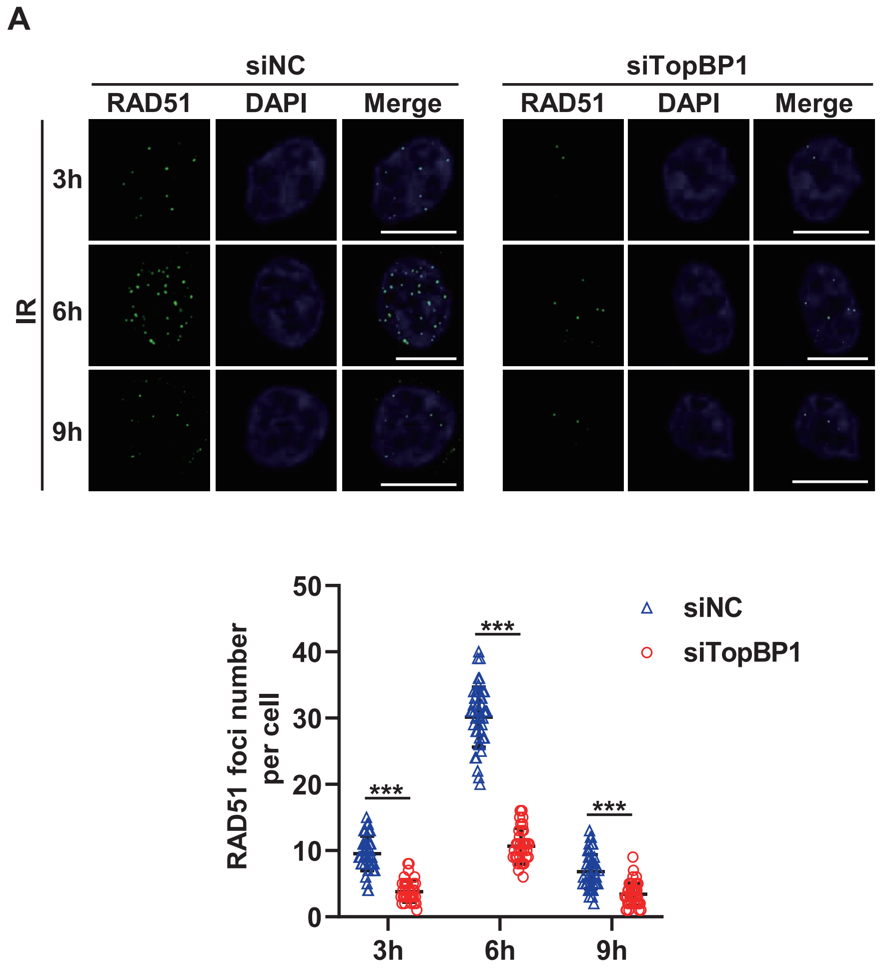

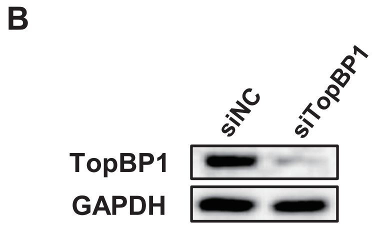

Supplemental Figure S15. TopBP1 regulates RAD51 recruitment. (A) TopBP1 depletion reduces IR-induced RAD51 foci formation. HCT116 cells were transfected with siNC or siTopBP1 oligos. 72 hours after transfection, HCT116 cells were treated with 5 Gy of IR. IRIF of RAD51 was examined by anti-RAD51 antibody at 3, 6 and 9 hours after IR (upper panels). The foci number in each cell was counted (lower panel). The bars represent the mean values ± SD (n = 50 from three independent experiments, per group). (**B**) siRNA treatment specifically down-regulates the expression of TopBP1. Two-tailed student's *t* test is used to determine statistical significance. ***, p ＜ 0.001, versus control groups. Scale bars, 10 μm.

Supplemental Figure S16

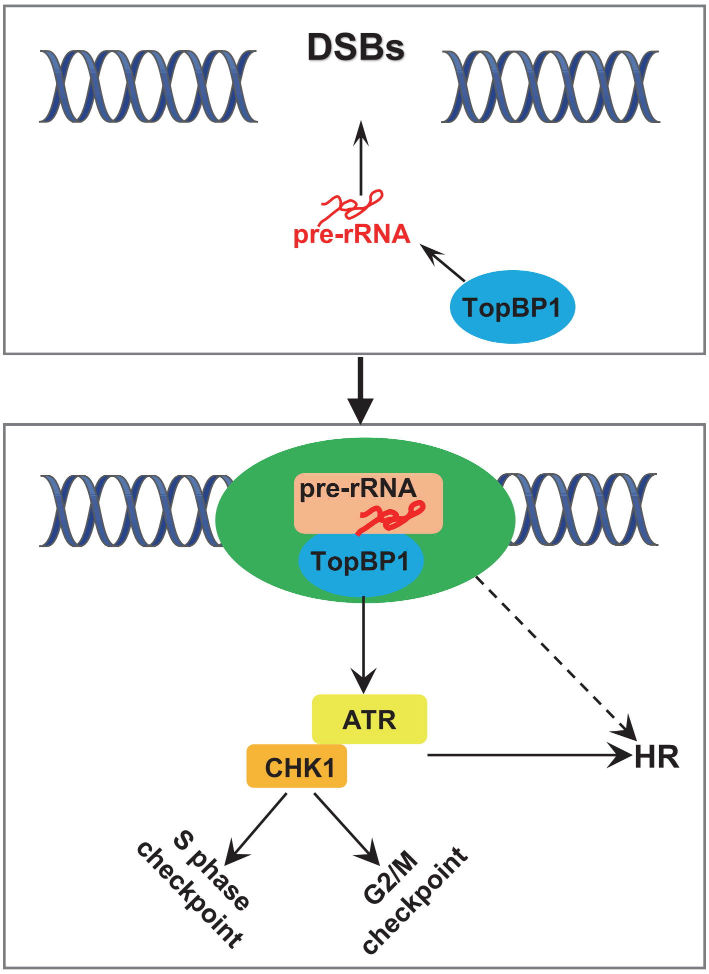

Supplemental Figure S16. A working model of pre-rRNA-mediated functions of TopBP1 during DNA damage response.

Supplemental Figure S17

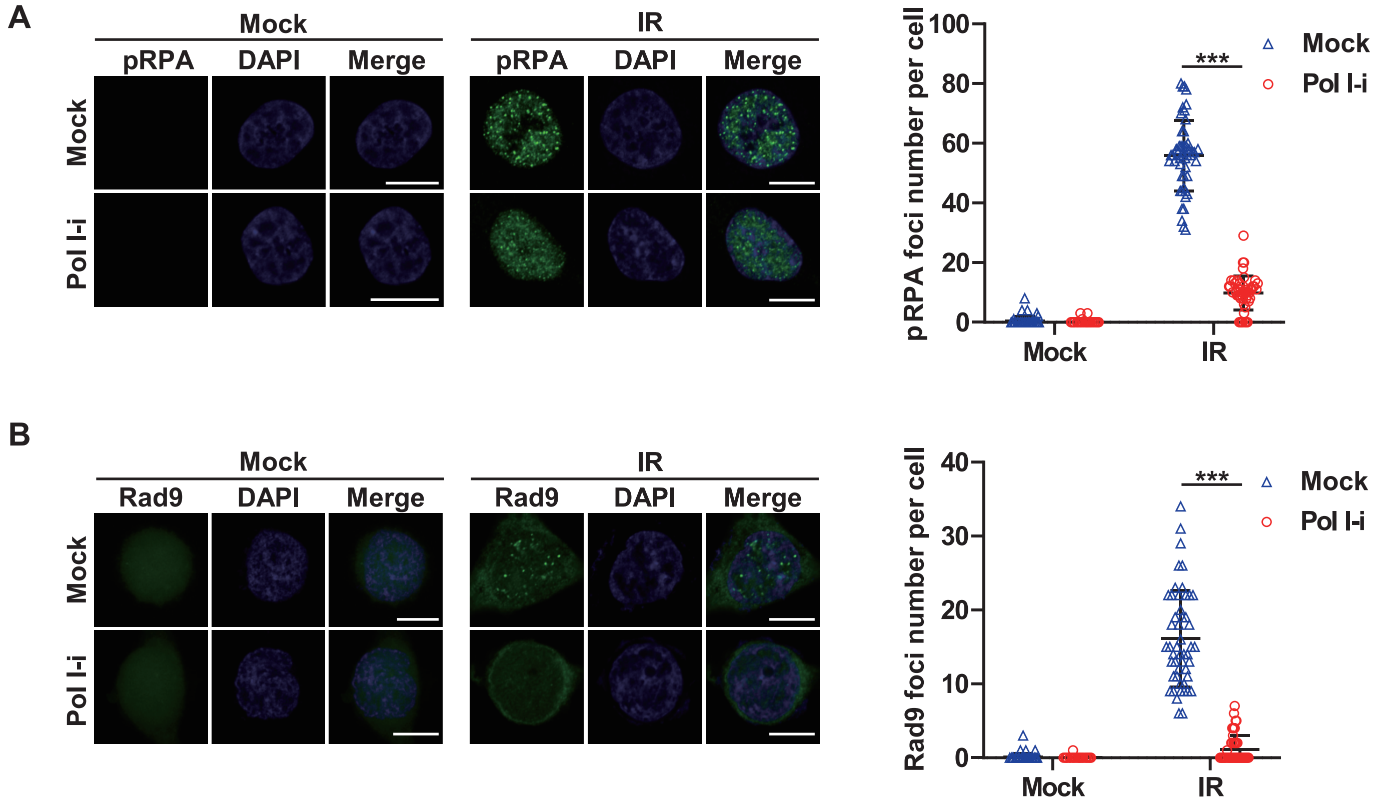

Supplemental Figure S17. RNA Pol I inhibitor treatment reduces the foci formation of RPA and Rad9. Following with or without Pol I treatment, HCT116 cells were treated with or without 10 Gy of IR. IRIF of RPA and Rad9 was examined by anti-pRPA (A) and anti-Rad9 antibodies (B) (left panels). The foci number in each cell was counted (right panels). The bars represent the mean values ± SD ($n = 50$ from three independent experiments, per group). Two-tailed student's t test is used to determine statistical significance. ***, $p < 0.001$, versus control groups. Scale bars, 10 μm.
